# Evidence that Vpu Modulates HIV-1 Gag-Envelope Interaction towards Envelope Incorporation and Infectivity in a Cell Type Dependent Manner

**DOI:** 10.1371/journal.pone.0061388

**Published:** 2013-04-16

**Authors:** Archana Gautam, Jayanta Bhattacharya

**Affiliations:** Department of Molecular Virology, National AIDS Research Institute, Bhosari, Pune, India; University of Nebraska Medical Center, United States of America

## Abstract

The HIV-1 Vpu is required for efficient virus particle release from the plasma membrane and intracellular CD4 degradation in infected cells. In the present study, we found that the loss of virus infectivity as a result of envelope (Env) incorporation defect caused by a Gag matrix (MA) mutation (L30E) was significantly alleviated by introducing a start codon mutation in *vpu*. Inactivation of Vpu partially restored the Env incorporation defect imposed by L30E substitution in MA. This effect was found to be comparable in cell types such as 293T, HeLa, NP2 and GHOST as well as in peripheral blood mononuclear cells (PBMC) and monocyte-derived macrophages (MDM). However, in HeLa cells BST-2 knockdown was found to further alleviate the effect of Vpu inactivation on infectivity of L30E mutant. Our data demonstrated that the impaired infectivity of virus particles due to Env incorporation defect caused by MA mutation was modulated by start codon mutation in Vpu.

## Introduction

The Human immunodeficiency virus type 1(HIV-1) Vpu plays an important role in HIV-1 pathogenesis via its interaction with various cellular proteins and overcoming antagonism by a restriction factor, BST-2 (also known as tetherin) [1]. Vpu, an 81-amino acid, phosphorylated oligomeric type I integral membrane protein is expressed from the same bicistronic mRNA that also encodes Env open reading frame [2], [3]. Translation of both the proteins, Vpu and Env occurs via leaky scanning by ribosomes [4] and mutations that disrupt *vpu* reading frame were reported to result in increased translation of *env* [5], [6]. Vpu performs two major roles during HIV-1 life cycle: enhancement of virus release from infected cells and selective degradation of CD4 receptor in the endoplasmic reticulum (ER) [7], [8]. Both Vpu and Env proteins of HIV-1 have profound effect on regulation of CD4 level during later stages of viral life cycle and are involved in the down-modulation of CD4 receptors from the surface of infected cells. During infection, the newly synthesized Env gp160 precursor retains CD4 in the ER by its high receptor binding affinity. As a result gp160-CD4 complexes not only prevent surface expression of CD4 but also block gp160 maturation and trafficking. Vpu regulates half-life of CD4 molecules complex to gp160 by inducing their degradation in the ER and releasing gp160, permitting its maturation, trafficking and incorporation into progeny virions [7], [9].

The two primary functions of Vpu are performed by two distinct structural domains (transmembrane and cytoplasmic domain). The transmembrane domain of Vpu is essential for enhanced virus release, and it does so by antagonizing the function of a cellular restriction factor, tetherin/BST-2 and thus enabling efficient release of progeny virions from infected cell [1], [10], [11]. The influence of Vpu on virus release is cell-type specific and this depends on the expression of BST-2 restriction factor, which is constitutively expressed in HeLa cells [10], [12], MOLT-4, Jurkat, primary T lymphocytes and macrophages [13]–[15], but is absent in permissive cells e.g., 293T, HT1080 and HOS [1], [10], [16].

Vpu plays an important role in HIV-1 assembly and budding process by regulating the intracellular trafficking of both Gag and Env proteins [11], [17], [18]. Vpu has been reported to influence Gag localization within the cellular milieu. A few studies have postulated a role of Gag matrix (MA) in Vpu-mediated particle release. The N-terminal of Gag MA is important for Vpu responsiveness during particle release and is crucial for Vpu function [19]. The functional interaction of Vpu with Gag through a tetratricopeptide repeat protein, UBP (Vpu-binding protein) has been shown and its role as an intermediate between Vpu and Gag was proposed to play an important role in virus assembly [20]. A recent study demonstrated that Vpu enhances virion release by preventing endosomal accumulation of Gag [11]. In addition to its effect on Gag trafficking, Vpu slows down the progress of Env glycoproteins along biosynthetic pathways, from ER and Golgi to plasma membrane [21]. However, Vpu does not interact directly with Env protein but is required for efficient cleavage of gp160 to gp120-gp41 subunits by destabilizing CD4-gp160 complexes in the ER and thereby allowing gp160 translocation to the Golgi apparatus [22], [23]. All these evidences are suggestive of a role of Vpu in subcellular trafficking of HIV-1 Gag and Env. At present, the mechanistic details of Vpu induced effect on Gag and Env are unclear.

On the other hand, Gag MA has been shown to have predominant effect on Envelope function. Previous studies demonstrated that specific amino acid substitutions in the Gag MA region abrogate incorporation of HIV-1 Env glycoproteins into virus particles [24]–[26] and redirects particle assembly to intracellular sites [27], [28]. Several studies suggest that the cytoplasmic domain of Env gp41 and the MA domain of Gag, together with various cellular cofactors, play a central role in HIV-1 assembly and specific alterations in the MA region of Gag affects Env incorporation onto virion particles, association with lipid rafts and thereby virion infectivity [24], [29]–[32]. While MA was shown to modulate Env assembly on mature virions, absence of Vpu has been shown to modulate Gag trafficking, assembly and infectivity besides modulation of particle release [11], [33]. Taken together, available information suggests interplay between Gag, Env and Vpu proteins and their cross-talk seems to be important for assembly and production of infectious virus particles.

In the present study we examined the effect of Vpu inactivation on Gag-Env interaction, particle release, Env incorporation and infectivity in different cell-types. We have examined whether Vpu plays any role in the MA-Env cross-talk, especially in context with Gag MA L30E mutation [25] which is known to diminish Env association with detergent resistant membranes (DRM), incorporation into virions and infectivity [32]. We found that inactivation of Vpu expression reduced the infectious potential of newly synthesized virions by reducing Env incorporation. Surprisingly, inactivation of Vpu significantly increased the infectious capability of Gag mutant (L30E) viruses by augmenting Env incorporation. Our data indicated that the Vpu modulates Env incorporation defects imposed by MA mutation through an unidentified mechanism.

## Results

### Construction of Vpu Start Codon Mutants, Gag Mutants and Envelope Deficient Backbones and their Intracellular Protein Expression

To analyze the effect of Vpu start codon mutation on virus release and infectivity, we used human cell lines where particle release was Vpu-dependent (HeLa cells) and independent (293T cells). In addition, we studied the effect of Vpu mutation on virus release and infectivity in NP2, GHOST and primary cells. To instigate the study we constructed versions of infectious pNL-AD8 HIV-1 molecular clone where single amino acid change (ATG to ATA) was introduced in *vpu* start codon. CCR5-tropic HIV-1 pNL-AD8 isolate was selected as it can replicate in both peripheral blood mononuclear cells (PBMC) and macrophages which are natural targets of HIV-1 *in vivo*. Molecular constructs possessing Gag MA mutation L30E (Leucine replaced with Glutamic acid at 30^th^ position) with and without Vpu start codon mutation were made to study the combined effect of both mutations on virus replication and infectivity. pNL-AD8 backbones lacking Env were also constructed by replacing *env* start codon with a stop codon (converting ATG to TAA) to study the consequences of Vpu and Gag mutations on primary envelopes derived from patients.

**Figure 1:**
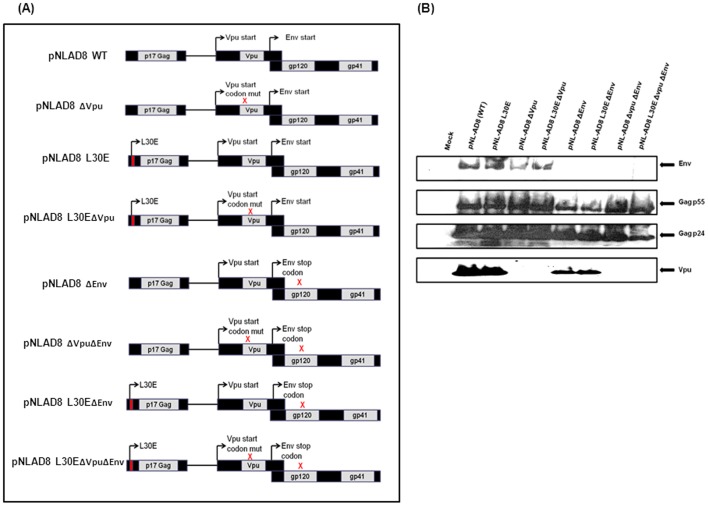
Construction of mutant clones. (**A**) Schematic representation of HIV-1 pNL-AD8 mutant clones used in the study. Diagram represent clones possessing Gag MA mutation (L30E), Vpu start codon mutation and HIV-1 pNL-AD8 Envelope deficient backbones possessing Gag MA (L30E) and Vpu start codon mutations. (**B**) Cell-associated viral gene expression of wild-type (WT), Gag and Vpu mutant clones and backbones carrying Env stop codon. 293T cells transfected with mutant plasmids were lyzed, cleared of cell-debris and nuclei by centrifugation and lysates were run in SDS-PAGE. Viral proteins were analyzed using anti-p24 (183-H12-5C), anti-gp41 (Chessie 8) antibody and anti-Vpu anti-serum. Note that substitution of ATG start codon of *vpu* with ATA abolished the expression of Vpu. Also, replacing Env start codon ATG with stop codon TAA abrogated the expression of pNL-AD8 Env. These Env deficient clones were further used in this study to make replication incompetent pseudotyped viruses from primary Envelopes.

Consequence of these mutations on intracellular expression of other viral proteins was determined by Western blot. Figure 1A illustrates position of mutations in the pNL-AD8 chimera. 293T cells were transfected with wild-type (WT) and mutant plasmids. At 48 h post-transfection, cell lysates were made and viral proteins were resolved in 10% SDS-PAGE followed by Western blotting (Figure 1B). Introduction of Vpu start codon mutation and Env stop codon mutation prevented expression of *vpu* and *env* gene. However, inactivation of *vpu* gene by introducing start codon mutation did not abrogate *env* gene expression and synthesis of intracellular Env protein. Also, mutation in *gag* MA (L30E) did not affect expression of Gag p24 and Gag p55 proteins.

### Effect of Vpu Inactivation on pNL-AD8 Virus Particle Release and Infectivity in Different Cell-types

**Figure 2:**
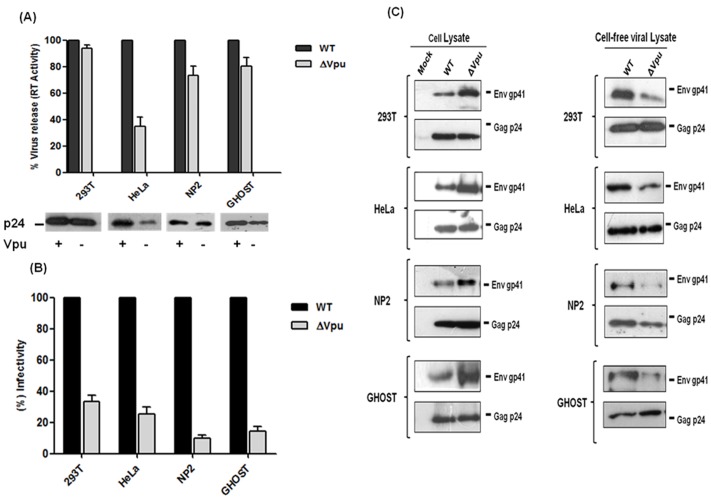
Differential effects of Vpu start codon mutation on release and infectivity of viruses from different cell types. Cell lines (293T, HeLa, NP2 and GHOST) were transfected with equal amount of wild-type pNL-AD8 (WT) plasmid and its Vpu defective variant (ΔVpu). At 48 h post-transfection, supernatants were harvested, clarified by centrifugation and filtered through 0.4 µm syringe filters. At the same time cell lysates were also prepared and cleared of cell debris and nuclei by centrifugation. (A) The amount of virus release in the supernatant was determined by measuring viral reverse transcriptase (RT) activity through RT-ELISA. Quantification of RT activity was done in duplicates, the average percent release and standard error was calculated considering WT release as 100%. Virus release was also evaluated by assessing viral Gag p24 protein in viral supernatants by 10% SDS-PAGE followed by Western blotting with HIV-1 Gag anti-p24 antibody (183-H12-5C). (B) Infectivity of ΔVpu viruses with respect to WT was determined in TZM-bl indicator cells. Clarified viral supernatants from transfected cells were serially diluted and added to the TZM-bl cells. At 48 h post-infection, infectivity was determined by measuring Luciferase activity as RLU (Relative Luminescence unit) using Luminometer. The average of RLU and standard error was calculated. Figure represents percent change in infectivity with respect to WT which was considered as 100%. (C) Western blots shown represent cell-associated and cell-free viral proteins. For blot of viral proteins, viral supernatants were concentrated by ultracentrifugation using 20% sucrose cushion and run in 10% SDS-PAGE. Before loading, viral lysates were normalized by RT value and equal RT values were subjected to SDS-PAGE. Viral proteins of both cell-associated and cell-free lysates were analyzed by using anti-p24 (183-H12-5C) and anti-gp41 (Chessie 8) antibodies. The release and infectivity experiments were performed in duplicates and the results shown are representative of three independent experiments.

Previously, Schubert *et al.* [5] reported that macrophage-tropic AD8 isolate carrying a mutation in its translation initiation codon of Vpu is capable of replicating in primary macrophages and PBMC with efficiency similar to its isogenic variant expressing Vpu. Later, Richards and Clapham [34] demonstrated that AD8 Env apparently failed to compensate for the lack of Vpu in primary macrophages. In both cases, viruses were made in two different producer cell types; while Schubert *et al.* [5] transduced HeLa cells, Richards and Clapham [34] used HEK 293T cells for virus production. To determine whether the effect of Vpu start codon mutation on virus particle release and infectivity of progeny virions vary among different producer cell types, we constructed a Vpu start codon mutant using full-length HIV-1 molecular clone, pNL-AD8 and transfected it in different cell types such as 293T, HeLa, NP2 and GHOST cells. Each of the cell lines were transfected with equal amounts of pNL-AD8 (WT) and its Vpu defective derivative, pNL-AD8ΔVpu. At 48 h post-transfection, supernatants containing virus were collected and virus release was quantified by measuring reverse transcriptase (RT) activity in the supernatant. As expected, RT ELISA result showed that virus release from HeLa cells was responsive to Vpu and we observed 60 percent reduction in the level of extracellular virions released from HeLa cells transfected with ΔVpu plasmid as compared to its wild-type counterpart (Figure 2A). In contrast, the effect of Vpu inactivation on virus particle release was not observed in 293T cells. Vpu mutant viruses from 293T cells were released at approximately wild-type level which is consistent with previous reported results [10], [12]. In case of NP2 and GHOST cells, the release of ΔVpu viruses was diminished by 25 and 20 percent, respectively in comparison to WT. Western blot analysis of cell-free virus particles showed that the release of HIV-1 particles (as detected by anti-p24 antibody) from HeLa cells was significantly impaired as compared to 293T, NP2 and GHOST cells (Figure 2A). These results confirmed previous findings that the ability of Vpu to enhance HIV-1 particle release is cell-type dependent.

Progeny viruses harvested from different producer cell types were tested for infectivity in TZM-bl indicator cells (Figure 2B). We found Vpu-defective viruses from 293T cells showed almost 60% reduction in infectivity whereas NP2 and GHOST derived viruses showed 90% reduction in infectivity potential. Results revealed that decrease in infectivity of ΔVpu viruses was always greater than the reduction in the total amount of virus released from transfected cells as measured by RT assay. The reduced infectivity potential of ΔVpu viruses from HeLa cells was result of reduced virus production, but in case of 293T, NP2 and GHOST cells despite having almost equivalent particle release from transfected cells we found reduction in infectivity potential of ΔVpu variant of pNL-AD8. Later, Western blot analysis of both cell-associated and cell-free viral proteins was performed. Equivalent quantities of viral lysates, as normalized by RT values were subjected to SDS-PAGE. Results revealed that reduction in infectivity potential of cell-free ΔVpu viruses from 293T, NP2 and GHOST cells was due to less Env (gp41) incorporation on progeny virions (Figure 2C). In case of HeLa cells also we observed less Env protein as compared to WT despite having equivalent p24. Further, blot of cell-associated viral proteins showed that the expression of intracellular Env was always more in ΔVpu as compared to WT in all cell-types including HeLa cells (Figure 2C). Similar observation was also reported previously by two groups that suggested increased translation of Env in absence of Vpu [5], [6]. Our data suggested that loss of Vpu not only affect virus release in cell-specific manner but also impairs virus infectivity by down-modulating Env incorporation on released virion particles.

### Introduction of Vpu Start Codon Mutation in pNL-AD8 with L30E Substitution Modulated Virus Infectivity and Particle Release

It is well documented that localization of Gag is influenced by Vpu. A few studies have suggested a role of HIV-1 Gag MA in Vpu-mediated virus particle release [19], [35], [36]. Their results demonstrated that re-distribution of HIV-1 Gag is Vpu-dependent and N-terminal MA domain of Gag is required for Vpu-responsiveness during particle release. Furthermore, Vpu expression has been shown to influence sub-cellular distribution of HIV-1 Gag [10], [11]. Based on observations that proposed the role of Gag MA in Vpu-mediated particle release, we next examined whether there is any alteration in Vpu mediated particle release and infectivity due to a specific point mutation in Gag MA (L30E) that was previously shown to severely diminish virus infectivity and Env incorporation [24], [25], [32] and whether it is dependent on cell-type.

**Figure 3:**
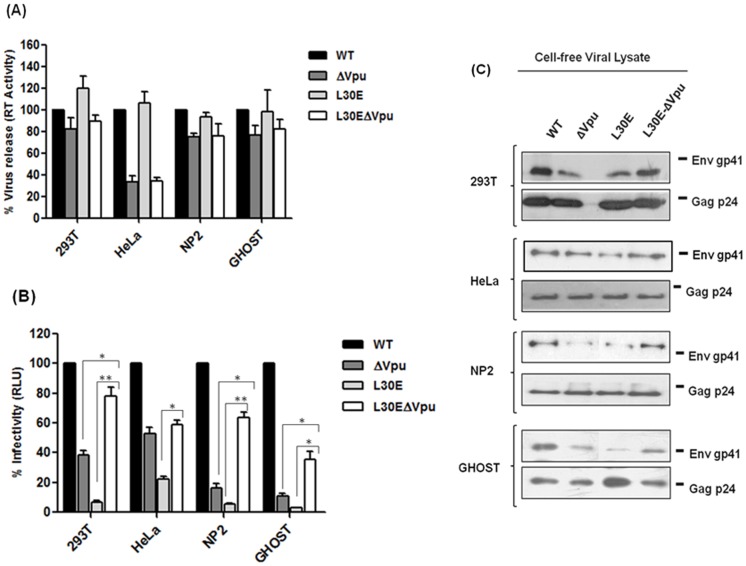
Vpu start codon mutation rescued infectivity of defective Gag MA mutant (L30E) viruses by modulating Env incorporation on released virions. (A) 293T, HeLa, NP2 and GHOST cells were transfected with four different plasmids (WT, ΔVpu, L30E and L30E-ΔVpu). WT and ΔVpu were used in each experiment of this study as control. At 48 h post-transfection, supernatants were collected and the level of virus release in supernatants was determined by RT ELISA. Percent RT activity and standard error was calculated with respect to WT assuming WT infectivity as 100%. (B) Infectivity of released virions was assessed in TZM-bl indicator cells. Viruses normalized for equal RT were added on TZM-bl cells. After 48 h, infectivity was determined by measuring Luciferase activity. The average of RLU and standard error was calculated. WT infectivity was considered as 100%. Significance in the increased infectivity of double mutant viruses (L30E-ΔVpu) was determined with respect to L30E and ΔVpu viruses using Student’s *t*-test: * means p<0.05 and ** means P<0.005. (C) Western blots shown represent cell-free viral proteins. Viral supernatants were concentrated by ultracentrifugation using 20% sucrose cushion and viral proteins were analyzed by western blotting using anti-p24 (183-H12-5C) and anti-gp41 (Chessie 8) antibodies. Virus release and infectivity experiments were performed in duplicates and the results shown are representative of three independent experiments. For western blot analysis, the viral supernatants of three independent experiments were pooled, concentrated, normalized for RT values and run in 10% SDS-PAGE gel.

To test this hypothesis, 293T, HeLa, NP2 and GHOST cells were transfected with pNL-AD8 (WT) plasmid and its mutant derivatives (ΔVpu, L30E and L30E-ΔVpu). Progeny virions obtained by transfection of these plasmids were examined and compared for infectivity and virus particle release. As shown in Figure 3A, we found that point mutation in Gag MA (L30E) did not affect particle release in any of the cell type used to prepare viruses. Instead, virus release of Gag L30E mutant was consistently greater than wild-type in 293T and HeLa cells and comparable in NP2 and GHOST cells. Significant reduction in particle release of ΔVpu and double mutant (L30E-ΔVpu) was monitored from HeLa cells as compared to 293T, NP2 and GHOST cells. Nonetheless, the release of L30E-ΔVpu viruses was similar to ΔVpu in HeLa cells but was significantly less than WT and L30E mutant.

We further tested infectivity of these viruses in TZM-bl indicator cells. We added equal virus particles normalized by equal RT values to infect TZM-bl cells. Infectious virion assay showed that L30E viruses displayed diminished infectivity irrespective of the cell type used for production of viruses and was in accordance with previous reports (Figure 3B) [25], [32]. In marked contrast, we found enhancement in the infectivity potential of double mutant virus (L30E-ΔVpu) indicating that inactivation of Vpu restored infectivity of L30E mutant viruses. This effect was observed in viruses from all cell-types. The enhancement in infectivity of L30E-ΔVpu viruses was less than wild-type but significantly higher than their L30E counterpart. The enhanced infectivity of double mutant (L30E-ΔVpu) varied between 3 to 7 fold among different cell types (293T, HeLa, NP2 and GHOST) in comparison to L30E mutant. In addition, we noticed significant difference between infectivity potential of ΔVpu and L30E-ΔVpu viruses from all cell types except HeLa cells where the infectivity of ΔVpu and L30E-ΔVpu was similar. This result suggests that the infectivity of defective L30E viruses was significantly alleviated by inactivation of *vpu* gene.

### Env Incorporation Defect Caused by Gag Matrix L30E Mutation was Partially Rescued by Vpu Start Codon Mutation

We further examined whether loss of Vpu expression has any association between modulations of infectivity of L30E viruses with Env incorporation on released virion particles. The supernatants containing progeny virions were harvested from transfected cells, 293T, HeLa, NP2 and GHOST, filtered through 0.45 µm syringe filters, concentrated by ultra-centrifugation using 20% sucrose in PBS and viral lysates were resolved in SDS-PAGE followed by Western blot with anti-gp41 and anti-p24 antibodies (Figure 3C). In order to determine the level of Env incorporation on released virions, equivalent quantities of viral lysates, as normalized by RT values were subjected to SDS-PAGE. As expected, the level of Env (gp41) on L30E viruses from all cell-types was significantly low as compared to wild-type. This decrease in the level of gp41 incorporated onto virions resulted in diminished infectivity of L30E viruses. In marked contrast, though the level of virus release of double mutant (L30E-ΔVpu) was less than the L30E variant but the amount of Env incorporation on released virion particles was comparatively more than L30E viruses, thus, contributing to enhanced infectivity of L30E-ΔVpu viruses (Figure 3C). Also, double mutant (L30E-ΔVpu) viruses possessed increased amount of Env than ΔVpu viruses from all cell types except HeLa cells where the amount of Env was similar in L30E-ΔVpu and ΔVpu and therefore was the reason of similar infectivity. These results suggested that inactivation of Vpu by introducing start codon mutation partially restored Env incorporation defect of Gag L30E mutants and hence improved their infectivity phenotype.

### Vpu Start Codon Mutation Restored Infectivity and Replication Defect of Gag (L30E) Mutant Viruses in PBMC and Monocyte-derived Macrophages

**Figure 4:**
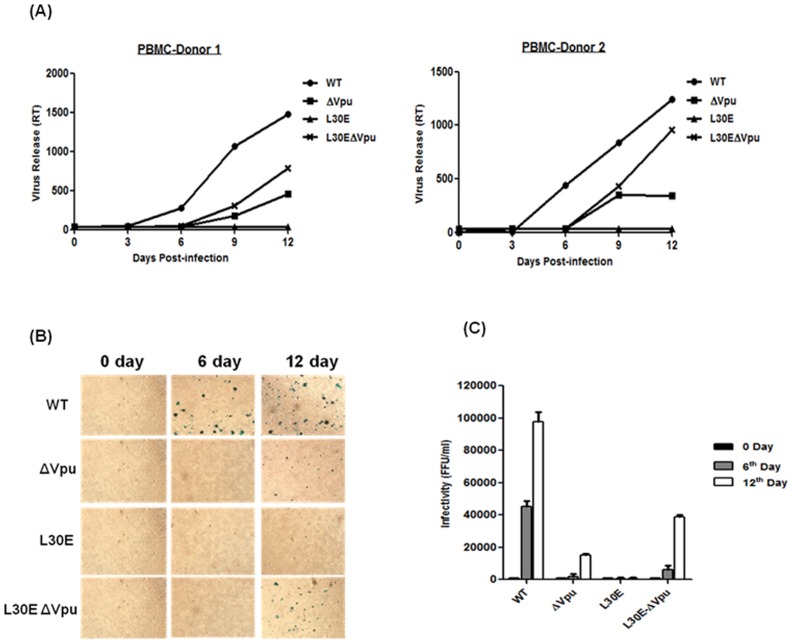
Virus propagation of Vpu and Gag L30E mutants in PBMC. Peripheral blood mononuclear cells (PBMC) were prepared from blood of healthy donors. The experiment was carried out in duplicate and in cells from two different donors and the results presented are averages of duplicate experiments. (A) PBMC obtained from two healthy seronegative donors were infected with equal focus-forming units (FFU) of WT and mutant (ΔVpu, L30E, L30E-ΔVpu) viruses pseudotyped with VSV-G. The amount of virus release in the supernatant was monitored and assessed up to 12 days by RT-ELISA. Virus release from infected PBMC was plotted as RT activity versus number of days. (B) Viral supernatants harvested from infected PBMC were tested for infectivity in TZM-bl indicator cells. Equal amount of viral supernatant (50 µL) was added on TZM-bl cells in duplicates. At 48 h post-infection, TZM-bl cells were fixed and stained with X-Gal substrate for β-galactosidase activity to give blue FFU. Figure shown represents infectivity of viruses harvested at three different days (0, 6^th^ and 12^th^ day). (C) Blue cells were counted as infectious units and calculated as FFU per milliliter and plotted as graph.

**Figure 5:**
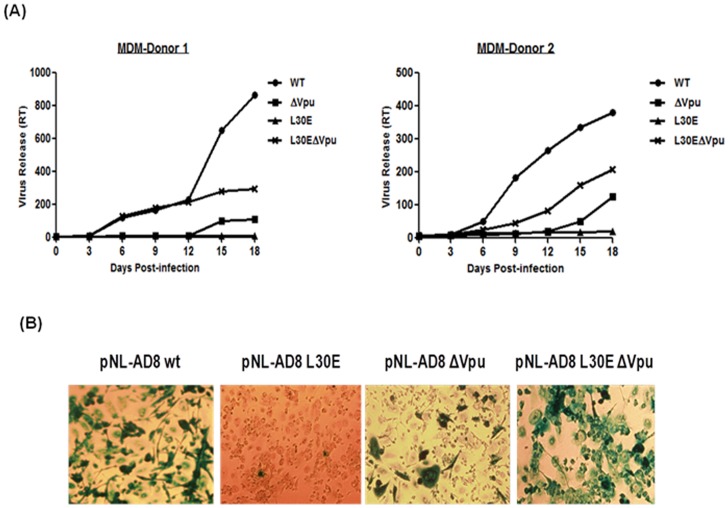
Virus propagation of Vpu and Gag L30E mutants in monocyte-derived macrophages. Macrophages were prepared from monocytes of two healthy seronegative individuals and infected with equal FFU of VSV-G pseudotyped viruses (WT and mutants). (A). Virus release was monitored for 18 days in MDM and RT activity was measured by RT-ELISA and plotted in a graph as RT activity versus number of days. (B) After 18 days, MDM infected with VSV-G pseudotyped viruses were fixed and permeabilized with 1∶1 acetone: methanol and infectivity was assessed by intracellular p24 immunostaining as described in material and methods. Blue cells were regarded as infected cells.

The importance and validity of results obtained under *in vitro* conditions cannot be fully substantiated until performed and observed in biologically relevant cells. Therefore, we further investigated whether inactivation of Vpu could alter the release and infectivity of Gag MA (L30E) mutants in primary cells that are natural targets of HIV-1 *in vivo*, such as peripheral blood mononuclear cells (PBMC) and monocyte-derived macrophages (MDM). To address this, PBMC and MDM were obtained from two HIV-1 seronegative healthy donors and infected with equal infectious units of VSV-G pseudotyped viruses (WT, ΔVpu, L30E and L30E-ΔVpu). Viruses were pseudotyped with VSV-G to ensure similar and efficient entry of mutant viruses in primary cells. Virus release and growth kinetics of mutant viruses was assessed by measuring RT activity in supernatants harvested from infected cells at different time points (Figure 4A and 5A). As expected and consistent with previous findings, Vpu-defective (ΔVpu) variant showed delayed onset of replication and poor infectivity in contrast to wild-type in both PBMC and MDM. L30E viruses which were defective in envelope incorporation replicated neither in PBMC nor in MDM in either of the donor. Interestingly, double mutant viruses (L30E-ΔVpu) replicated efficiently in both PBMC and MDM, though their release was 50% less as compared to wild-type. The poor ability of Vpu deficient viruses to replicate in primary macrophages was consistent with that of the findings of Richards and Clapham [34].

Furthermore, cell supernatants containing viruses collected from infected PBMC at different time points were used to infect TZM-bl indicator cells to examine infectious virus particles (Figure 4B). Equal amount of viral supernatant was added to TZM-bl cells. After 48 h, cells were fixed and stained for β-galactosidase activity using X-gal and infectivity was assessed by examining focus forming units (FFU) as a measure of intracellular p24 expression. FFU per millilitre were counted and plotted in a graph (Figure 4B). As evident from Figure 4B, at day 12, the infectivity of L30E-ΔVpu double mutant viruses was found to be significantly more than L30E and ΔVpu mutant viruses. Similar observations were made with MDM (Figure 5A). After 18 days of infection, MDM were fixed and immunostained for p24 antigen. The number of infected MDM could not be counted as majority of cells were fused and formed syncytia (Figure 5B). Overall, our findings show that the abrogation of virus growth and infectivity due to Gag L30E mutation was rescued by Vpu inactivation in primary cells also.

In order to check whether the effect of Vpu inactivation on virion incorporation is specific to HIV-1 Env we tested the infectivity potential of VSV-G pseudotyped viruses in GHOST-CD4-CXCR4 cells as described in material and methods. We found infectivity of the VSV-G pseudotyped viruses (all WT and mutants) was unaffected and all Vpu and Gag L30E mutants were showing infectivity similar to WT (Figure S1). This confirmed that the effect of Vpu inactivation on Env incorporation is HIV-1 Env specific.

### Effect of Vpu Start Codon Mutation in Restoring Gag L30E Defect Verified in Unrelated Patient-derived Envelopes of Diverse Origin

**Figure 6:**
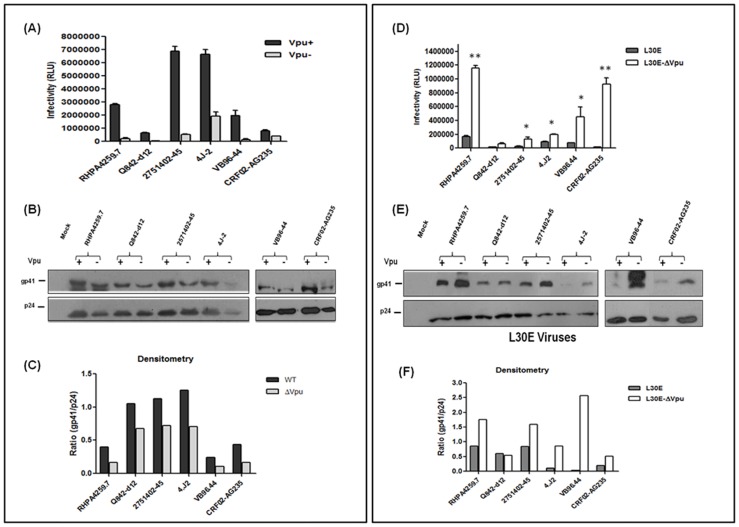
Effect of Vpu start codon mutation and Gag L30E mutation on infectivity and Env incorporation of primary viruses of diverse origin. Viruses were produced by co-transfection of 293T cells with combinations of Env deficient backbone plasmids containing Gag (L30E) or Vpu mutation and Env plasmids of patient origin. At 48 h post-transfection, supernatants were harvested and infectivity of viruses was determined in TZM-bl cells by measuring RLU. Average of RLU and standard error was calculated and plotted as a graph. Infectivity experiments were performed in duplicates and the results shown are representative of three independent experiments. Viral supernatants were concentrated by ultracentrifugation using 20% sucrose cushion, normalized for RT values and equal quantities were subjected to 10% SDS-PAGE gel, followed by Western blot with anti-p24 (183-H12-5C) and anti-gp41 (Chessie 8) antibody. Western blots shown represent gp41 Env on released virion particles. Viral p24 and gp41 band intensities were determined with Image J software (NIH) and ratio of gp41 to p24 was plotted in a graph to determine Env incorporation. (A) Infectivity of primary viruses in presence (+) and absence (−) of Vpu. (B) Western blot analysis of Vpu^+^ and Vpu*^−^* primary viruses. (C) Densitometry analysis of band intensities of Vpu^+^ and Vpu*^−^* viral p24 and gp41 proteins. Ratio of gp41/p24 represents the amount of Env (gp41) incorporation on virions. (D) Infectivity of primary viruses carrying Gag L30E mutation in presence and absence of Vpu. Significance level of improved infectivity of double mutant viruses (L30E-ΔVpu) was determined with respect to viruses possessing Gag mutation (L30E) using Student’s *t*-test: * means p<0.05 and ** means P<0.005. (E) Western blot analysis of L30E and L30E-ΔVpu (double mutant) primary viruses. (F) Densitometry analysis of L30E and L30E-ΔVpu viral proteins, p24 and gp41. Ratio of gp41/p24 represents the amount of Env (gp41) incorporation on virions.

We next verified whether the effect of Vpu start codon mutation on L30E mediated infectivity defect was specific to lab-adapted pNL-AD8 construct or equally effective in primary HIV-1 envelopes of diverse origin derived from patients. We tested following primary envelopes from different clades [RHPA4279.8 (clade B), Q842.d12 (clade A), 2571402.45 (clade C, acute category), 4.J2 (clade C, early category), VB96.44 (clade C, AIDS category), and CRF02.AG235 (A/G recombinant)]. To generate single-cycle replication incompetent viruses expressing patient envelopes, we made *env* deficient backbones using pNL-AD8 as vector and introducing stop codon in place of Env start codon (replacing ATG with TAA) through PCR mutagenesis as described in materials and methods. Four pNL-AD8*env^−^* backbones were constructed with Vpu and Gag MA mutations as illustrated in Figure 1A. Using these *env^−^* backbones, 293T cells were co-transfected with patient Env plasmids in order to generate single-round infectious virions expressing patient envelopes. At 48 h post-transfection, viral supernatants were collected, clarified and tested for infectivity in TZM-bl cells in serial dilutions in duplicates. Infectivity was determined by measuring Luciferase activity (Figure 6A). As shown in Figure 6A, infectivity of all primary envelopes was reduced in absence of Vpu; however when Gag L30E mutation was accompanied with Vpu start codon mutation, significant improvement in infectivity of patient envelopes was observed (Figure 6D). To further elucidate any modulation in Env incorporation on virus particles, viral supernatants were concentrated by ultra-centrifuge and RT activity was measured. Equivalent quantities of cell free Env-pseudotyped viral lysates, as normalized by RT values were subjected to SDS-PAGE followed by Western blot using anti-gp41 and anti-p24 antibodies. As shown in Figure 6E, modest restoration of Env incorporation was noticed in case of all the primary Envs and was supported by densitometry analysis of band intensities of gp41 and p24 (Figure 6F). This further suggested that the effect of Vpu inactivation in restoring envelope incorporation defect of L30E viruses is universal and irrespective of viral strain, however the level of effect may vary from strain to strain. Taken together, this data clearly indicates that inactivation of *vpu* by start codon mutation was found beneficial in rescuing the defect of Gag L30E mutation.

### Cellular Distribution of HIV-1 Envelope in HeLa Cells

**Figure 7:**
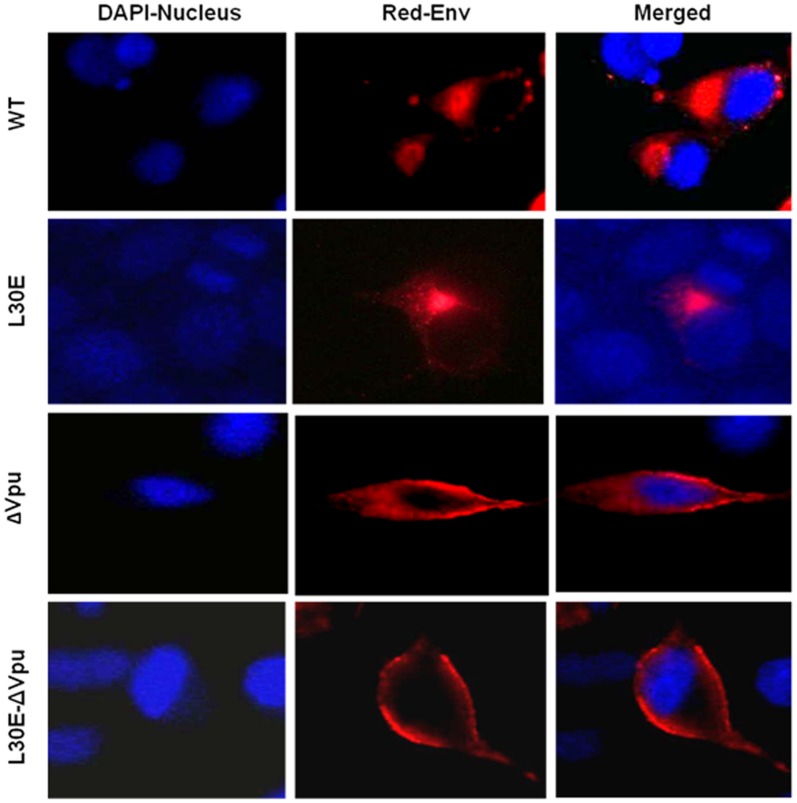
Vpu inactivation affected distribution of Env in HeLa cells. HeLa cells were transfected with WT and mutant plasmids (L30E, ΔVpu, L30E-ΔVpu). At 24 h post-transfection, cells were washed, fixed, permeabilized and immunolabeled with anti-Env antibody, 2F5. Nuclei were stained using ProLong Anti-fade DAPI (Molecular Probes, Invitrogen). Cells were visualized using Olympus IX51 microscope at 60X oil immersion objective. Scale bar: 10 µm.

RT-ELISA and Western blot analysis revealed that inactivation of *vpu* by start codon mutation had profound effect on virus release from HeLa cells in contrast to other cell types used in the study. Also when L30E mutation was accompanied with ΔVpu, we did not observe much difference in the infectivity of double mutant and ΔVpu viruses from HeLa cells. We thought that this might be due to restricted particle release from HeLa cells in the absence of Vpu. We further investigated distribution of Env in absence and presence of Vpu in HeLa cells by immunofluorescence and compared it with 293T cells. Cells were transfected on cover slips with four different constructs (WT, ΔVpu, L30E, L30E-ΔVpu) and 36 h after transfection, cells were fixed, permeabilized and immunolabeled with anti-Env monoclonal antibody 2F5. We found that in HeLa cells, if expressed in presence of Vpu (WT), majority of Env protein was distributed throughout the cytoplasm and some was also localized at discrete sites at the plasma membrane (Figure 7). But in the absence of Vpu (ΔVpu and L30E-ΔVpu), a significant fraction of Env was found localized at plasma membrane. The Env of Gag L30E mutant was detected mainly near the perinuclear compartment and away from the plasma membrane. In contrast, in 293T cells we found Env was diffusely present throughout the cytoplasm in WT, ΔVpu and L30E-ΔVpu, (data not shown). These results raised speculation about the role of host restriction factor modulating this phenomenon in HeLa cells.

### Expression of BST-2 in HeLa Cells Vary from Other Cell-types

**Figure 8:**
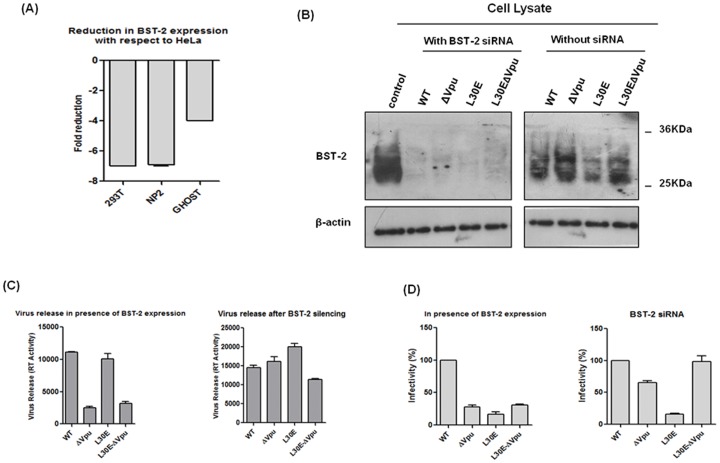
Knockdown of endogenous BST-2 in HeLa cells increases production of infectious HIV-1 particles. (A) Relative Real-time analysis of endogenous expression of BST-2 was done in three cell-types (293T, NP2, GHOST) using HeLa as a control. Plot represents fold reduction in the mRNA expression of BST-2 in different cell-types with respect to HeLa. (B) Western blotting was used to determine the change in expression of BST-2 in HeLa cells in presence and absence of BST-2 siRNA; Control was lysate of cells transfected with control siRNA and β-actin was used as a loading control. (C) Levels of HIV-1 particles in supernatants obtained from transfected HeLa cells (with and without BST-2 siRNA) were quantified by measuring viral RT activity. (D) Infectivity titre of WT and mutant viruses produced from transfected HeLa cells (with and without BST-2 siRNA) was determined in TZM-bl cells by Luciferase assay.

It is now known that Vpu promotes enhanced virus release by counteracting host restriction factor, BST-2, however the level of BST-2 expression differ in cell types [1], [37]. We therefore analysed the mRNA expression level of BST-2 in four different producer cell-types (293T, HeLa, NP2 and GHOST) through quantitative real-time PCR as described in material and methods. The variation in expression level of BST-2 among different cell-types was detected by comparative threshold cycle (2*^−^* ^ΔΔCt^ method) using β-actin as an endogenous internal control. Results revealed that the BST-2 mRNA expression was 4–7 folds more abundant in HeLa cells as compared to other cells, 293T, NP2 and GHOST (Figure 8A). This data and the immunofluorescence data together indicate that BST-2 host factor might be responsible for restricted release of ΔVpu and L30E-ΔVpu viruses in HeLa cells.

### RNAi-mediated Knockdown of BST-2 not only Enhanced Virion Release of Vpu Deleted Viruses from HeLa Cells but also Increased the Infectivity of Double Mutant Viruses

We next examined whether BST-2 was responsible for accumulation of Env protein at plasma membrane in HeLa cells and thus diminished release and infectivity of ΔVpu viruses. To answer this question, we used siRNA oligonucleotides to deplete endogenous BST-2 in HeLa cells and then transfected siRNA treated cells with WT, ΔVpu, L30E and L30E-ΔVpu DNA. BST-2 depletion was confirmed by Western blot analysis of transfected cell lysates (Figure 8B). Results showed that knockdown of endogenous BST-2 led to more than 3 fold increases in the total amount of virus release of ΔVpu and L30E-ΔVpu as quantified by RT ELISA (Figure 8C). Infectivity in TZM-bl cells using equal RT units showed 40% increase in infectivity level of L30E-ΔVpu viruses as compared to ΔVpu viruses (Figure 8D). This result confirmed that variation in infectivity phenotype of double mutant viruses produced from HeLa cells resulted from the accumulation of Env at the plasma membrane due to presence of host restriction factor BST-2. Our results indicated that disrupting BST-2 expression by siRNA in HeLa cells not only restored the release of ΔVpu viruses but further alleviated the infectivity of double mutant viruses (L30E-ΔVpu) and this rescue of infectivity of the L30E mutant by Vpu inactivation is most likely due to enhanced envelope incorporation.

## Discussion

In the present study, we have investigated the consequences of Vpu start codon mutation on HIV-1 release and infectivity. It is well documented that HIV-1 Vpu plays a vital role in viral pathogenesis via enhancement of virus release and degradation of intracellular CD4 thus, liberating Env gp160 for further processing and maturation. The obscure role of Vpu in Gag and Env trafficking demonstrated by a few studies prompted us to investigate the effect of Vpu start codon mutation on Env and Gag function. We have investigated the impact of Vpu start codon mutation on release and infectivity of viruses produced from different cell-types including primary cells. Furthermore, we have studied the impact of Vpu inactivation on release and infectivity of Gag MA mutant (L30E).

Previously, it was reported that HIV-1 AD8 isolate, which naturally carries *vpu* start codon mutation is capable of replicating in primary cells with efficiency similar to its wild-type counterpart [5]. Later, Richards and Clapham [34] demonstrated that AD8 Env apparently failed to compensate for the lack of Vpu in primary macrophages. Here, we found that inactivation of *vpu* by introduction of start codon mutation in CCR5-tropic HIV-1 pNL-AD8 diminished the release and infectivity of the virus in cell-type dependent manner. We show that Vpu-deleted viruses displayed impaired release from HeLa cells but not from other three cell-types (293T, NP2 and GHOST). These findings suggest cell-type differences in virus release pathway of HeLa cells as compared to other cells used in the study. Our data was supported by previous findings that proposed the presence of host restriction factor in HeLa cells which keeps enveloped virions tethered to the cell surface [1], [11], [12], [38]. When infectivity of ΔVpu viruses was assessed in TZM-bl cells, we observed adverse effect of Vpu inactivation on virus infectivity irrespective of the cell line used for virus production. Despite having equivalent release of both ΔVpu and wild-type viruses from 293T, NP2 and GHOST, we found 60–80% reduction in infectivity. In HeLa cells, low infectivity of ΔVpu viruses resulted from reduced virus release. Similarly, Vpu-deleted viruses displayed poor replication in primary cells, PBMC and MDM and showed delayed onset of replication in comparison to wild-type. Our results in monocyte-derived macrophages were consistent with the report demonstrated by Richards and Clapham [34].

A recent study proposed that Vpu affects trafficking of Env and in the absence of Vpu, Env is frequently found within cytoplasmic accumulations that are clathrin-coated and contain Gag [11]. Other studies showed that this cytoplasmic compartment contained markers suggestive of virion assembly. It contain proteolytically processed Gag p17 [11], a marker for mature virion: CD63 and CD81, marker for membrane domain associated with viral assembly [39], and AIP1/ALIX, a cellular cofactor for viral budding [40]–[42]. These findings suggested that these cytoplasmic structures are late endosomes and likely contain mature virions, either assembled within these structures or assembled at the plasma membrane and subsequently internalized. In our study, Western blot analysis of released virions from different cell-types revealed that in contrast to wild-type, ΔVpu viruses possessed less Env (gp41) and thus was the reason of reduced infectivity of ΔVpu mutant. The effect of Vpu inactivation on Env incorporation was more severe on viruses produced from 293T, NP2 and GHOST as compared to HeLa cells. In primary cells also ΔVpu viruses replicated with reduced efficiency which could also be explained by the fact that Vpu interferes with the formation of gp160-CD4 complexes in dose-dependent manner and promote gp160 processing [7], [9]. In the absence of Vpu, newly synthesized Env forms complexes with CD4 molecule and thus prevents its maturation, trafficking and incorporation into released virions. In agreement with previous studies we also found that, inactivation or deletion of Vpu by introducing start codon mutation resulted in enhanced cellular expression of Env [5], [6]. Possible explanation for this occurrence is the phenomenon of leaky scanning of ribosomes which is common in retroviruses. This process occurs when the first initiation codon of a bicistronic mRNA is unavailable, as a result ribosomal subunit bypass this site and slips to second nearby AUG site to start initiation. Overall, our data substantiated previous findings, illustrating the role of Vpu in Env synthesis and processing, and in addition supports the notion that Vpu is required for optimal HIV-1 infectivity and pathogenesis.

Role of HIV-1 Vpu in intracellular Gag trafficking has been postulated earlier but the site of Vpu function is still not known and how Vpu influences Gag and Env assembly is yet to be established. Several studies have shown Vpu as an important determinant of Gag accumulation in endosomes [10], [33], [43]. The steady state localization of Gag is influenced by Vpu; if co-expressed with Vpu, majority of Gag stays at the cell surface, but in the absence of Vpu a significant fraction of Gag is subsequently endocytosed into endosome-like compartment from the cell surface [33]. It was previously reported that Vpu most likely controls targeting of Gag in infected T cells and macrophages because deletion of *vpu* gene results in accumulation of virions into intracellular compartments [44], [45]. A few contradictory studies have postulated a role of Gag MA in Vpu-mediated particle release. It was proposed that N-terminal of Gag MA is important for Vpu responsiveness during particle release and is crucial for Vpu function [19]. Another study reported functional interaction of Vpu with Gag through a tetratricopeptide repeat protein, UBP (Vpu-binding protein) [20], [46]. Furthermore, Vpu has been shown to influence both Gag and Env localization [11], [20], [35]. All these evidences suggest a role of Vpu in HIV-1 Gag and Env trafficking and assembly. Intriguingly, by contrast to what was observed with Gag L30E viruses, in which infectivity of viruses was severely abrogated due to reduced Env incorporation, we observed significant restoration of infectivity of L30E viruses in absence of Vpu (Figure 3B). We show here that the inactivation of Vpu by start codon mutation rescued the infectivity defect of Gag mutant (L30E) viruses by enhancing Env incorporation on released virion particles. In addition we observed significant variation between infectivity of ΔVpu and L30E-ΔVpu mutants from 293T, NP2 and GHOST cells. Moreover, in contrast to L30E and ΔVpu mutant viruses, double-mutant (L30E-ΔVpu) viruses replicated efficiently in primary cells (PBMC and MDM) also. Initially, we failed to observe difference in infectivity potential of ΔVpu and L30E-ΔVpu mutants from HeLa cells. Later, Immunofluorescence data confirmed that in HeLa cells, majority of Env protein of ΔVpu viruses was localized at the cell periphery and some was present in the cytoplasm (Figure 7), whereas in 293T cells diffused distribution of Env throughout the cytoplasm was observed in ΔVpu constructs which was no different from WT (data not shown). This indicated cell-type variation in HIV assembly.

Next we tested expression level of a host restriction factor, BST-2, which is constitutively expressed in HeLa cells and compared with other cell lines. qRT-PCR experiments revealed that BST-2 mRNA expression was 4–7 folds more abundant in HeLa cells as compared to other cell types tested in this study (Figure 8). Knockdown of endogenous BST-2 revealed that silencing of BST-2 in HeLa cells not only increased the production of ΔVpu viruses but also enhanced the infectivity of double mutant (L30E-ΔVpu) viruses compared to L30E and ΔVpu viruses. We anticipate that viruses that come out of cell by overcoming the host restriction factor (BST-2/tetherin) might have alteration in their overall morphology such as number of glycoprotein spikes as compared to viruses that buds out normally. That’s why we did not observed much difference in the infectivity of ΔVpu and double mutant viruses from HeLa cells that expresses tetherin/BST-2 constitutively.

Immunostaining data showed increased syncytium formation by double mutant (L30E-ΔVpu) in MDM as compared to WT and other mutants (Figure 5B). Low level of BST-2 has been detected in macrophages [37] and therefore could be the reason of Env accumulation at cell surface causing cell-cell fusion or syncytium formation. Finally, our data corroborated the finding that the restoration of infectivity of L30E viruses by inactivation of Vpu is a universal phenomenon that is occurring in lab-adapted as well as primary cells. We also confirmed that this phenomenon was not the characteristic feature of HIV-1 AD8 Env, as similar results were obtained after co-transfection of pNL-AD8Δ*env* backbone with primary Envs of different clades (Figure 6). One possible explanation that could be offered for this phenomenon is that apart from CD4 degradation, Vpu may also be acting at the plasma membrane and participating in the assembly events of the virus. Recently the presence of Vpu in lipid rafts, considered to be potential sites of virus assembly and release was shown [47]. The N-terminal MA domain of Gag is critical for membrane binding and was reported to be Vpu-responsive [19]. It may be possible that at plasma membrane Vpu regulates the incorporation of Env on budding virus particles and this activity of Vpu is dependent on MA domain of Gag. Therefore, specific mutation in the MA domain of Gag might alter Vpu’s ability to regulate Env trafficking to virus budding sites.

Various studies suggested that in absence of Vpu, virion particles have been found both at plasma membrane and in internal vesicles similar to late endosomes. It was demonstrated that these virion particles that are found in internal vesicles are fully matured virus particles that have been targeted to these sites via en route from plasma membrane. It was clearly demonstrated by Pelchen-Mathhews *et.al.*, that HIV buds from internal vesicles, also known as micro-vesicular bodies (MVBs) from monocyte-derived macrophages [45]. Their immunolabeling experiments suggested that virions observed in intracellular vesicles possessed significant amount of Env as observed through anti-Env antibody staining, indicating that Env is enriched on these budding virions. But how these Envs are sorted into late endosomes budding structures is not known. The presence of high amount of Env on virions assembling into late endosomes suggests that these particles are likely to be infectious. Another study by Joshi *et al*., [48] also suggested that intracellular compartments (e.g., MVBs) are capable of serving as sites for productive assembly in MDMs and T cells. Further studies are necessary to understand how MA domain of Gag is important for regulatory role of Vpu in HIV-1 Gag and Env trafficking. It will be important to determine whether Vpu diverts Gag and Env to the site of virus assembly or Gag MA determines the site of action of Vpu to participate in formation of infectious virions. Overall, our results demonstrated Vpu as a regulator of HIV-1 Gag assembly and Env incorporation.

Our study provided new insight into the molecular mechanism regulating HIV-1 infectivity, Env incorporation during virus assembly and production of infectious virion particles from cell lines and physically relevant cell-types, like PBMC and monocyte-derived macrophages. We speculate that two viral proteins, Gag and Vpu together with various cellular factors play an important role in determining the site of virus release and formation of infectious virus particles. Further studies are required to understand the detailed mechanism of Gag and Env trafficking and to delineate the intracellular transport steps affected by Vpu at the time of HIV-1 assembly. Such information would likely provide comprehensive understanding on the mechanism of assembly and Env incorporation during HIV-1 morphogenesis.

## Materials and Methods

### Cell Lines, Antibodies and Plasmids

Human embryonic kidney (HEK) 293T cells were obtained from American Type Culture Collection (ATCC), TZM-bl and HeLa cells were obtained from NIH AIDS Research Reagents and Reference Program (NIH ARRRP); while GHOST-parental, NP2-parental and GHOST-CXCR4 cell lines [49] were kindly provided by Dr Paul Clapham, UMASS Medical School, Worcester, MA, USA. Anti-gp41 hybridoma (Chessie 8), mouse anti-p24 (183-H12-5C) monoclonal antibody (mAb), anti-gp41 mAb 2F5 and rabbit anti-Vpu antiserum were obtained from NIH ARRRP. Secondary antibody conjugated to horse radish peroxidase (HRP) were obtained from Thermo Scientific, Inc., while goat-anti-mouse IgG conjugated to β-galactosidase used for immunostaining was obtained from Southern Biotech Inc. RHPA4259.7 and 2571402-45 envelope plasmids were kindly provided by Dr. David Montefiori, Duke University while pNL-AD8 [50], Q842-d12 [51] and CRF02_AG235 plasmids were obtained from NIH ARRRP. VB-96.44 and 4.J2 are clade C patient derived Envs were described previously [52], [53].

### Construction of Molecular Clones and their Protein Expression

Mutations introduced in all plasmids were accomplished by primer overlap strategy. The point substitution (L30E) in *gag* matrix (MA) was introduced by PCR using internal primers carrying specific L30E substitution and outer primers having unique 5′ BssHII and 3′ SphI restriction sites. The *p17* gene was amplified from pNL-AD8 using above primers with high fidelity Platinum Taq DNA Polymerase (Invitrogen, Carlsbad, California). The PCR amplified fragment was digested with BssHII and SphI restriction enzymes and ligated back into pNL-AD8 vector digested with same restriction enzymes (BssHII and SphI) using T4 DNA Ligase (New England Biolabs, Inc.) to give rise to Gag MA mutant, pNL-AD8L30E. To introduce Vpu start codon mutation in pNL-AD8 wild-type (WT) and pNL-AD8L30E, internal primer pair was designed containing a single nucleotide substitution replacing AT**G** (methionine) with AT**A** (isoleucine) and outer primer pair containing 5′ EcoRI and 3′ BamHI restriction sites. Cloning of these mutants was accomplished as described above. These molecular constructs have been referred as ΔVpu and L30E-ΔVpu throughout the study. Molecular variants of pNL-AD8 (WT), L30E, ΔVpu and L30E-ΔVpu were further constructed by converting the *env* start codon into a stop codon (ATG to TAA) to give rise WTΔEnv, L30EΔEnv, ΔVpuΔEnv and L30E-ΔVpuΔEnv backbones, respectively. These four molecular clones were used as backbone in subsequent experiments to generate pseudotyped primary viruses. All clones were confirmed by sequencing for presence of mutations.

### Cell Culture and Transfection

All cell lines were propagated in Dulbecco’s modified Eagle’s medium (DMEM) (Invitrogen, Carlsbad, California) supplemented with 10% FBS and 100 µg of penicillin-streptomycin/ml (Invitrogen). Cells were grown at 37°C with 5% CO_2_ in humidified incubator. 293T, HeLa, NP2-parental and GHOST-parental cells were seeded in 6-well trays (4×10^5^ cells per well) 24 h prior to transfection. Cells were transfected with 4 µg of plasmid DNA using Lipofectamine 2000 according to manufacturer’s protocol (Invitrogen). After 6–8 h, medium of transfected cells was replaced with fresh 10% DMEM. Viruses were harvested after 48 h, clarified by centrifugation at 2000 rpm for 5 min at 4°C and filtered through 0.45 µm pore size syringe filters to remove residual cells and debris. Harvested viruses were aliquoted and stored at −80°C freezer for future use.

For production of pseudotyped primary viruses, 293T cells were co-transfected with 4 µg of ΔEnv backbone plasmids and 2 µg of patient Env plasmid using Lipofectamine. Viruses were harvested after 48 h as described above.

### RT ELISA

Viral supernatants harvested from transfected cells (293T, HeLa, NP2 and GHOST) were assessed for virus release by measuring reverse transcriptase (RT) activity using commercially available RT-ELISA kit (CavidiTech, Uppsala, Sweden) according to the manufacturer’s protocol.

### Infectivity Assay

The infectivity potential of viruses was determined on TZM-bl cells. Cells were plated in 96-well plate at a density of 10,000 cells/well and subsequently infected with 10-fold serial dilutions of viruses harvested from transfected cells. After 3 h, fresh growth medium was added on top of infected cells. Cells were incubated for 48 h at 37°C in 5% CO_2_ incubator. The virus titers were determined by performing Luciferase assay (Promega) according to manufacturer’s recommendation using Luminometer (Victor 4, Perkin Elmer, Inc.).

### Preparation of Pseudotyped (VSV-G+) Viruses

A vesicular stomatitis virus (VSV-G) expression vector was co-transfected with following plasmids (WT, ΔVpu, L30E and L30E-ΔVpu) in 3∶1 ratio in 293T cells using Lipofectamine method. Progeny pseudovirions were harvested at 48 h, clarified by centrifugation (2000 rpm for 5 min), filtered by 0.45 µm pore size syringe filter, aliquoted, and stored at −80°C. Pseudotyped viruses were tested for infectivity in GHOST-CD4-CXCR4 cells which under normal conditions are not infected by CCR5 using viruses. Viruses were serially diluted and equal amount of supernatant was added on GHOST-CD4-CXCR4 cell plated in 96-well titre plate (10,000 cells/well) one day before infection. 48 h post-infection, GHOST cells were fixed with acetone-methanol (1∶1 ratio) and immunostained for p24 antigen as described previously by Peters *et al* [54]. Blue cells were counted as infectious units and their average was calculated per millilitre (Figure S1). Equal FFUs of these viruses was then later used to infect PBMCs and MDM.

### PBMC and Monocyte-derived Macrophage Isolation and Culture

Following approvals of the National AIDS Research Institute (NARI) Review Board and NARI Ethics Committee and after obtaining written informed consent, PBMC were isolated from 20 ml whole blood of two healthy, HIV-1 seronegative individuals by using Ficoll-Hypaque method and resuspended in 5 ml RPMI 1640 (Invitrogen, Inc.), supplemented with 10% FBS and antibiotic mix. PBMC were stimulated for 2 days with phytohemagglutinin (PHA: 5 µg/ml; Sigma-Aldrich, St. Louis, MO). After 2 days, PBMC were maintained in 10% RPMI supplemented with human interleukin-2 (IL-2∶20 U/ml; Roche).

For monocyte-derived macrophages (MDM) preparation, monocytes were isolated from PBMC using monocyte-isolation kit (Invitrogen) according to the protocol described by manufacturer. Monocytes obtained by using this kit were 99% pure. Purified cells were resuspended in 6-well plate in 2 ml 10% DMEM containing MCSF (macrophage colony stimulating factor from Sigma-Aldrich: 5 µg/ml) and incubated at 37°C in CO_2_ incubator for 2 days. After 48 h, media of adherent cells was replaced with fresh 10% DMEM and cells were incubated for another 6 days. After 6 days, MDM were obtained.

### Infection of PBMC and MDM

PBMC (2×10^6^ cells) were resuspended in 500 µl 10% RPMI-IL2 medium in 48-well plate. PBMC were infected with 1000 focus forming units (FFU) of VSV-G pseudotyped viruses. Following 3 h adsorption, cells were washed twice with plain RPMI and resuspended in 500 µl 10% RPMI-IL2 medium. Infected PBMC were maintained at 37°C in 5% CO_2_ incubator. Every 3-day time interval, 100 µl culture supernatant was collected up to 12 days, cells were fed with equal amount of fresh medium and the amount of virus release in the collected supernatant was analysed by measuring RT activity using RT-ELISA kit (Cavidi Tech).

For infection of MDM, adherent macrophages were washed with plain DMEM, removed using Versene (Invitrogen) for 3–5 min and scraped gently with a cell scraper. Macrophages were resuspended in 10% DMEM, plated in 96-well tray (20,000 cells/well) in 200 µl DMEM and incubated overnight at 37°C in CO_2_ incubator. Next day, macrophages were infected with 100 FFU of VSV-G pseudotyped viruses. After 3 h of adsorption, macrophages were washed twice with plain DMEM to remove residual cell-free virus, and resuspended in 200 µl 10% DMEM. Every 3-day time interval, 50 µl culture supernatant was collected up to 18 days, cells were fed with equal amount of fresh medium and amount of virus release was analysed by measuring RT activity in the collected supernatants using RT-ELISA kit.

### Western Blotting

To check the intracellular expression of viral proteins, transfected cells were lysed in ice-cold lysis buffer (0.5% Triton X-100, 50 mM Tris-HCl pH 7.5, 150 mM NaCl, 5 mM EDTA) supplemented with protease inhibitor cocktail (Sigma-Aldrich) and cellular debris was removed by centrifugation at 20,000×g for 20 min in a microfuge at 4°C. Equivalent amounts of cell lysates were subjected to 10% SDS-PAGE and transferred to PVDF membrane (Millipore) by electro blotting for 1 hour in a Bio-Rad semi-dry Trans Blot cell (Bio-Rad, Hercules, CA). The membrane was blocked for 1 h with 5% non-fat dry milk and incubated overnight with anti-gp41 monoclonal antibody (mAb) Chessie 8 (1∶100 dilution of hybridoma supernatant), anti-p24 mAb 183-H12-5C (1∶3000 dilution) and rabbit anti-Vpu antiserum (1∶5,000 dilutions). Membrane was washed and probed with horseradish peroxidase (HRP)-conjugated anti-mouse or anti-rabbit secondary antibodies (1∶5,000 dilution) (Thermo Scientific) for 1 h and protein bands were detected using the Super Signal West Dura Substrate from Thermo Scientific, Pierce.

### Env incorporation Assay

To estimate the amount of Env incorporation onto virus particles, viral supernatants harvested from transfected cells were centrifuged at low speed, passed through 0.45 µm pore size syringe filters to remove cell debris and virus particles were pelleted using 20% sucrose in PBS at 30,000×g for 2 h at 4°C in an ultracentrifuge (Beckman Coulter, Fullerton, CA). Virus pellets were reconstituted in 1X PBS, RT activity was determined and equivalent amount of viral lysates were resolved in 10% SDS-PAGE followed by Western blot onto PVDF membrane. Membrane was probed with anti-gp41 mAb Chessie 8 (1∶100 dilution of hybridoma supernatant) and anti-p24 mAb 183-H12-5C (1∶3000 dilution) followed by incubation with (HRP)-conjugated anti-mouse secondary antibody (1∶5,000 dilution).

### Immunofluorescence Microscopy

293T and HeLa cells (1×10^5^) were plated in 6 well tissue culture plate containing cover slips and transiently transfected with 1 µg of WT pNL-AD8 plasmid and its Gag and Vpu variants. At 24 h post-transfection, cells were fixed with 4% paraformaldehyde and permeabilized for 10 min using 0.1% Triton X-100. The cells were blocked with 1% bovine serum albumin in PBS and stained with specific antibody against Env protein (2G12 at 0.5 µg/ml) at room temperature. After 1 h, cells were washed thrice with PBS, followed by incubation with anti-human secondary antibody conjugated with Cy3 (1 µg/ml; Southern Biotech) for 30 min at room temperature. The cells were washed thrice with PBS and cover slips were mounted on slides using ProLong Antifade DAPI mounting media (Molecular Probes, Invitrogen, UK). Images were captured by fluorescence microscope (Olympus IXF1, Olympus, Inc.) using 60X oil immersion objective and images were processed using Cell-F software (Olympus Inc.).

### Quantitative Real Time PCR (qRT-PCR)

Total mRNA was extracted from cells using RNeasy (Qiagen, Inc.) and 500 ng of RNA was used to generate cDNA using Superscript III reverse transcriptase (Invitrogen, Inc.). Specific primers for BST-2 (CCGTCCTGCTCGGCTTT [forward] and CCGCTCAGAACTGATGAGATCA [reverse] described previously by Mansouri *et al* [55] ) and β-actin (GGCGACGAGGCCCAGA [forward] and CGATTTCCCGCTCGGC [reverse] [56] ), were used for qRT-PCR. Transcript levels were determined by qRT-PCR using SYBR green dye incorporation (Invitrogen) in an ABI 7900HT sequence detection system. The comparative threshold cycle method (2-ΔΔCt method) was used to quantify the difference in BST-2 gene expression between different cell-types, using β-actin as an endogenous internal control.

### siRNA Mediated BST-2 Depletion

Seeded HeLa cells were transfected with a pool of three siRNA targeting BST-2 (each 3 nmol) or a control siRNA pool (10 µM) using siRNA transfecting reagents (Santa Cruz Biotech, CA) according to manufacturer’s protocol. After 24 h, siRNA treated cells were transfected with plasmids using Lipofectamine method. At 48 h post-transfection, viral supernatant was harvested and cell-lysates were prepared using RIPA buffer (Sigma-Aldrich) containing protease inhibitor cocktail.

### Statistical Analysis

Significance between two sets of values was determined by Student’s t test using Graph Pad prism software and P values were obtained.

## Supporting Information

Figure S1 **Infectivity of VSV-G pseudotyped viruses.** VSV-G pseudotyped viruses were made by co-transfecting 293T cells with VSV-G plasmid and following plasmids (WT, ΔVpu, L30E and L30E-Δvpu). Progeny pseudovirions were harvested at 48 h, clarified by centrifugation (2000 rpm for 5 min), filtered through 0.45 µm pore size syringe filter and tested for infectivity in GHOST-CD4-CXCR4 cells. (A) Viruses were serially diluted and equal amount of supernatant was added on GHOST-CD4-CXCR4 cell plated in 96-well plate (10,000 cells/well). As positive infectivity control, pNL4.3 virus without VSV-G was added and as negative control pNL-AD8 virus without VSV-G was added on GHOST-CD4-CXCR4 cells. 48 h post-infection, GHOST cells were fixed with acetone-methanol (1∶1 ratio) and immunostained for p24 antigen. Blue cells represent focus forming units (FFU) or infectious units. (B) Blue cells were counted as infectious units, their average was calculated per millilitre and plotted in a graph.
(TIF)
