# Expression of Concern: Evidence that Vpu Modulates HIV-1 Gag-Envelope Interaction towards Envelope Incorporation and Infectivity in a Cell Type Dependent Manner

**DOI:** 10.1371/journal.pone.0301303

**Published:** 2024-03-26

**Authors:** The PLOS ONE Editors

After this article [1] was published, concerns were raised about results presented in Figs 1–4, 6 and 8. Specifically:

- The Fig 2A 293T panel and the Fig 2C Cell-free viral lysate 293T Gagp24 panel appear similar when flipped.
- The Fig 2A GHOST panel and the Fig 2C Cell-free viral lysate NP2 Gagp24 panel appear similar.

- There appear to be vertical discontinuities in the backgrounds between lanes 2 and 3 in the NP2 and GHOST Envgp41 panels in Fig 3C.
- All 0 day panels in Fig 4B appear similar to each other.
- There are vertical discontinuities in the backgrounds between lanes 2 and 3 in the panels on the right in Fig 6B and 6E.
- The Fig 8B right β-actin panel and lanes 2–5 of the Fig 8B left β-actin panel appear similar when flipped.
- In all western blot panels in Fig 1B, and in the Cell Lysate HeLa panel in Fig 2C, the blot image background details are not visible in the published figures.

In response to queries about the experiments in Fig 2A and 2C, the corresponding author stated that the 293T panel in Fig 2A and the Cell-free viral lysate 293T Gagp24 panel in Fig 2C are intentionally the same and present the same results. The corresponding author also acknowledged that they inadvertently duplicated the Gag p24 blot of NP2 in Fig 2C under GHOST in Fig 2A. A corrected version of Fig 2 is provided here where the GHOST panel in Fig 2A has been replaced. The underlying blot for the Fig 2A GHOST panel and the Fig 2C Cell-free viral lysate NP2 Gagp24 panel is in S1 File. The underlying blot for the Fig 2A 293T panel and the Fig 2C Cell-free viral lysate 293T Gagp24 is no longer available. The *PLOS ONE* Editors consider the similarity between the published Fig 2A GHOST panel and the Fig 2C Cell-free viral lysate NP2 Gagp24 panel resolved, and consider the similarity between the Fig 2A 293T panel and the Fig 2C Cell-free viral lysate 293T Gagp24 panel unresolved.

In response to queries about the experiments in Fig 4B, the corresponding author stated that at 0 days, TZM-bl indicator cells are expected to be similar as they are the same cell type and no infection was observed. A corrected version of Fig 4 is provided here where the WT, ΔVpu and L30E 0 day panels have been replaced. The underlying images for Fig 4B are in S1–S3 Files. The *PLOS ONE* Editors consider the above concerns about results presented in Fig 4B resolved.

In response to queries about the experiments in Fig 6B and 6E, the corresponding author stated that due to a large number of samples, some were run separately in different gels at different times but the data was shown together. An updated version of Fig 6 is provided here where splice lines between lanes 2 and 3 in the right panels in Fig 6B and 6E are denoted with vertical black lines. The underlying blots for the right panels in Fig 6B and lanes 1–2 of the right panels in Fig 6E are in S1 File. The underlying blot for lanes 3–4 of the right panels in Fig 6E are no longer available. The *PLOS ONE* Editors consider the above concerns about results presented in Fig 6B resolved, and the above concerns about results presented in Fig 6E unresolved.

Individual-level data and original uncropped image files for some charts and panels in Figs 1–4, 6 and 8 are provided here in S1 File. These uncropped images resolve the above concerns about the backgrounds in Fig 1B but do not resolve the above concern about the background in the Cell Lysate HeLa panel in Fig 2C. The corresponding author stated that the remainder of the data underlying this article [1] are no longer available.

In light of the unresolved concerns in Figs 2–3, 6 and 8, the extent of image issues in this article, and the unavailability of some original underlying data for the figures of concern, the *PLOS ONE* Editors issue this Expression of Concern.

**Fig 2:**
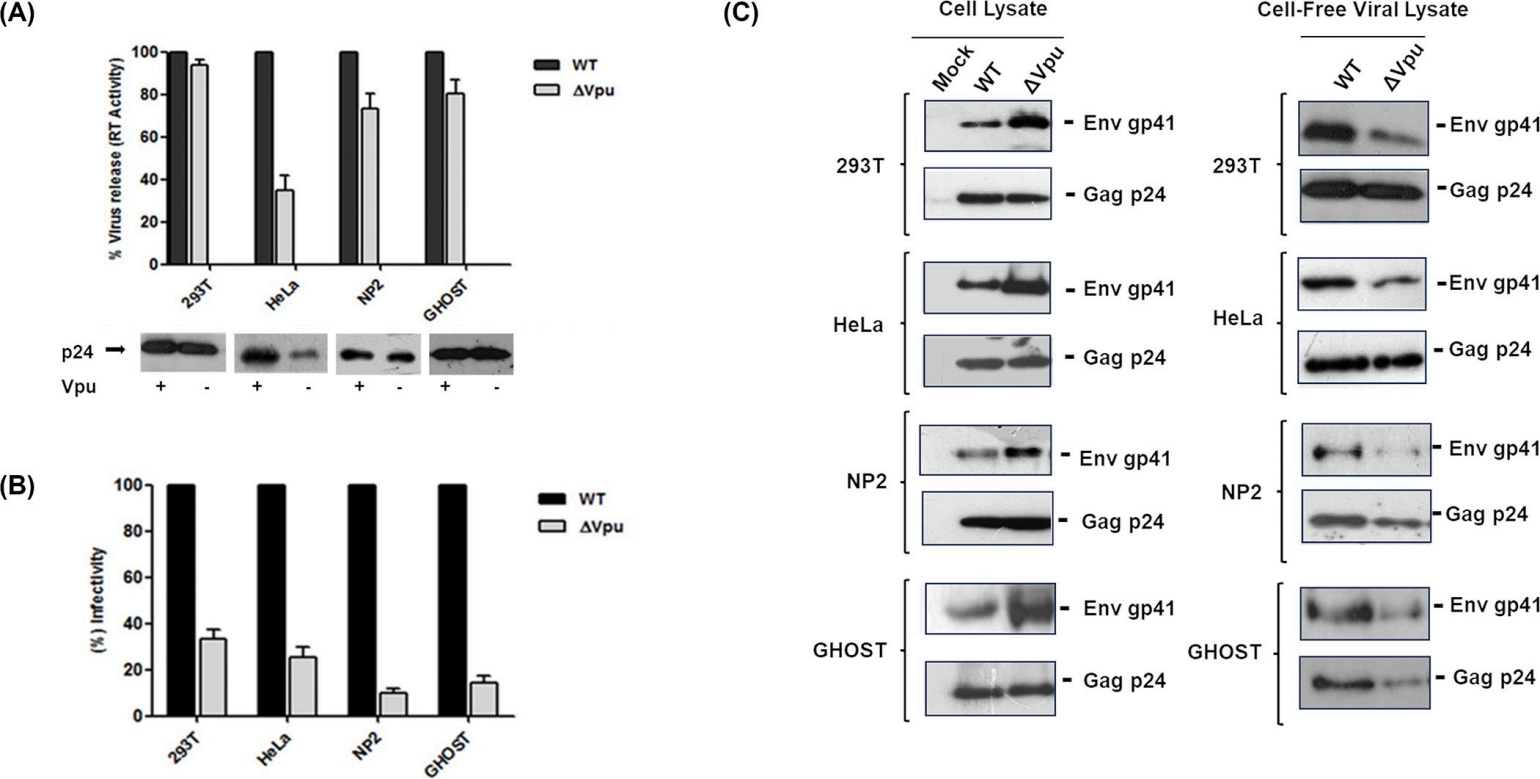
Differential effects of Vpu start codon mutation on release and infectivity of viruses from different cell types. Cell lines (293T, HeLa, NP2 and GHOST) were transfected with equal amount of wild-type pNL-AD8 (WT) plasmid and its Vpu defective variant (ΔVpu). At 48 h post-transfection, supernatants were harvested, clarified by centrifugation and filtered through 0.4 μm syringe filters. At the same time cell lysates were also prepared and cleared of cell debris and nuclei by centrifugation. (A) The amount of virus release in the supernatant was determined by measuring viral reverse transcriptase (RT) activity through RT-ELISA. Quantification of RT activity was done in duplicates, the average percent release and standard error was calculated considering WT release as 100%. Virus release was also evaluated by assessing viral Gag p24 protein in viral supernatants by 10% SDS-PAGE followed by Western blotting with HIV-1 Gag anti-p24 antibody (183-H12-5C). (B) Infectivity of ΔVpu viruses with respect to WT was determined in TZM-bl indicator cells. Clarified viral supernatants from transfected cells were serially diluted and added to the TZMbl cells. At 48 h post-infection, infectivity was determined by measuring Luciferase activity as RLU (Relative Luminescence unit) using Luminometer. The average of RLU and standard error was calculated. Figure represents percent change in infectivity with respect to WT which was considered as 100%. (C) Western blots shown represent cell-associated and cell-free viral proteins. For blot of viral proteins, viral supernatants were concentrated by ultracentrifugation using 20% sucrose cushion and run in 10% SDS-PAGE. Before loading, viral lysates were normalized by RT value and equal RT values were subjected to SDS-PAGE. Viral proteins of both cell-associated and cell-free lysates were analyzed by using anti-p24 (183-H12-5C) and antigp41 (Chessie 8) antibodies. The release and infectivity experiments were performed in duplicates and the results shown are representative of three independent experiments.

**Fig 4:**
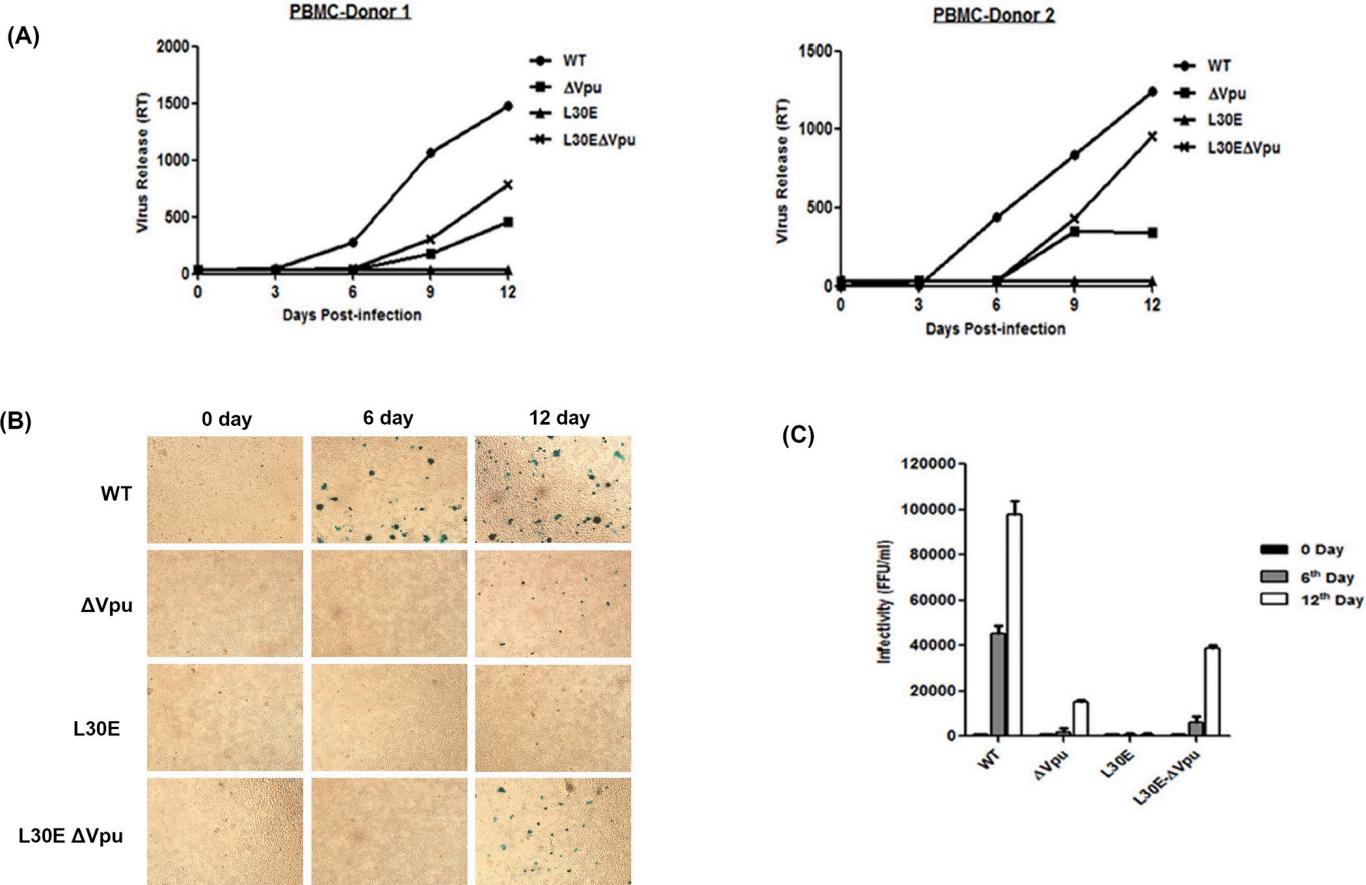
Virus propagation of Vpu and Gag L30E mutants in PBMC. Peripheral blood mononuclear cells (PBMC) were prepared from blood of healthy donors. The experiment was carried out in duplicate and in cells from two different donors and the results presented are averages of duplicate experiments. (A) PBMC obtained from two healthy seronegative donors were infected with equal focus-forming units (FFU) of WT and mutant (ΔVpu, L30E, L30E-ΔVpu) viruses pseudotyped with VSV-G. The amount of virus release in the supernatant was monitored and assessed up to 12 days by RT-ELISA. Virus release from infected PBMC was plotted as RT activity versus number of days. (B) Viral supernatants harvested from infected PBMC were tested for infectivity in TZM-bl indicator cells. Equal amount of viral supernatant (50 μL) was added on TZM-bl cells in duplicates. At 48 h post-infection, TZM-bl cells were fixed and stained with X-Gal substrate for β-galactosidase activity to give blue FFU. Figure shown represents infectivity of viruses harvested at three different days (0, 6^th^ and 12^th^ day). (C) Blue cells were counted as infectious units and calculated as FFU per milliliter and plotted as graph.

**Fig 6:**
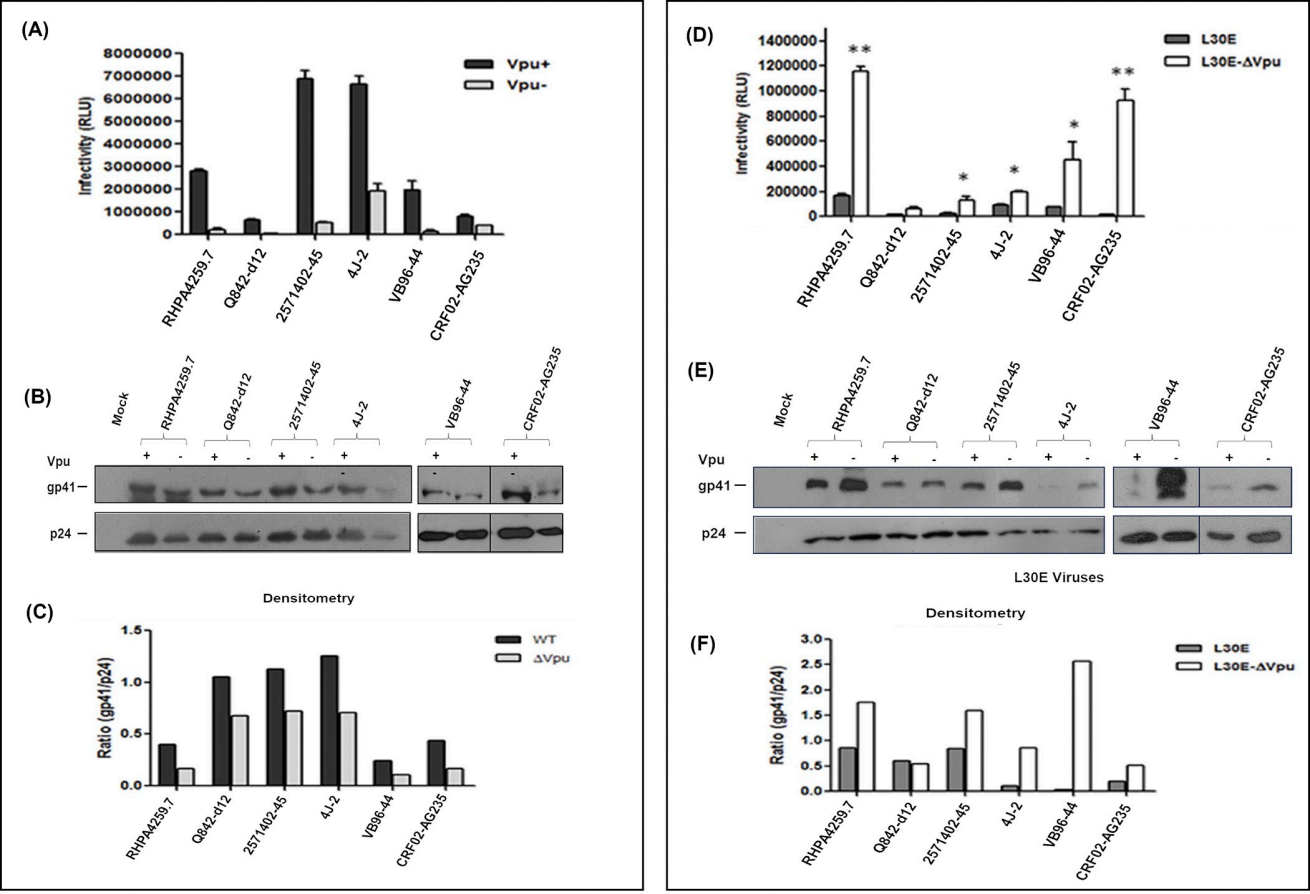
Effect of Vpu start codon mutation and Gag L30E mutation on infectivity and Env incorporation of primary viruses of diverse origin. Viruses were produced by co-transfection of 293T cells with combinations of Env deleted backbones plasmids containing Gag (L30E) or Vpu mutation and Env plasmids of patient origin. At 48 h post-transfection, supernatants were harvested and infectivity of viruses was determined in TZM-bl cells by measuring RLU. Average of RLU and standard error was calculated and plotted as graph. Infectivity experiments were performed in duplicates. Viral supernatants of independent experiments were pooled and concentrated by ultracentrifugation using 20% sucrose cushion, normalized for RT values and equal quantities were subjected to 10% SDS-PAGE gel, followed by Western blot with anti-p24 (183-H12-5C) and anti-gp41 (Chessie 8) antibody. Western blots shown represent gp41 Env on released virion particles. Viral p24 and gp41 band intensities were determined with Image J software (NIH) and ratio of gp41 to p24 were plotted in a graph to determine Env incorporation. (A) Infectivity of primary viruses in presence (+) and absence (-) of Vpu. (B) Western blot analysis of Vpu^+^ and Vpu^-^ primary viruses. The vertical black lines in the blot indicates spliced bands. (C) Densitometry analysis of band intensities of Vpu^+^ and Vpu^-^ viral p24 and gp41 proteins. Ratio of gp41/p24 represents the amount of Env (gp41) incorporation on virions. (D) Infectivity of primary viruses carrying Gag L30E mutation in presence and absence of Vpu. Significance level of improved infectivity of double mutant viruses (L30E-ΔVpu) was determined with respect to viruses possessing only Gag mutation (L30E) using Student’s *t*-test: * means p< 0.05 and ** means P< 0.005. (E) Western blot analysis of L30E and L30E-ΔVpu (double mutant) primary viruses. The vertical black lines in the blot indicates spliced bands. (F) Densitometry analysis of L30E and L30E-ΔVpu viral proteins, p24 and gp41. Ratio of gp41/p24 represents the amount of Env (gp41) incorporation on virions.

## Supporting information

S1 File Individual-level data for the charts in Figs 3A–3B, 4A and 4C and 8C, some underlying images from the time of the original experiments supporting Figs 1B, 2A, 2C, 4B, 6B, 6E and 8B, and underlying replicate blots from later repeat experiments for Fig 8B. (ZIP)

S2 File Underlying original image for the WT 12 day panel in Fig 4B. (JPG)

S3 File Underlying original image for the L30E ΔVpu 12 day panel in Fig 4B. (JPG)
