# Supplementary figures and images for: Evidence that Vpu Modulates HIV-1 Gag-Envelope Interaction towards Envelope Incorporation and Infectivity in a Cell Type Dependent Manner
Source: PLoS One. 2013 Apr 16;8(4):e61388. doi: 10.1371/journal.pone.0061388 (PMC3628852; doi:10.1371/journal.pone.0061388)

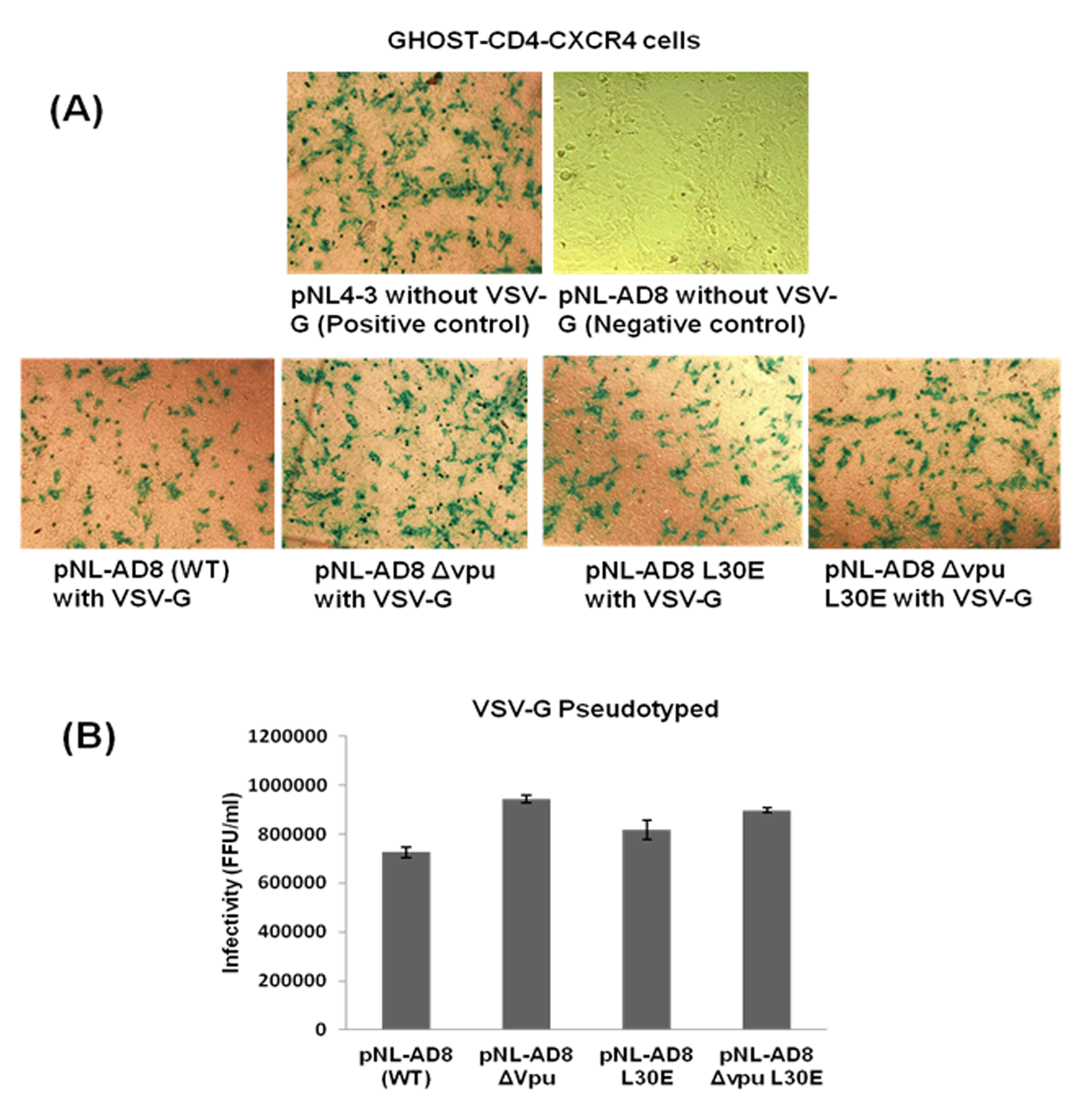

Supplement: Figure S1 — Infectivity of VSV-G pseudotyped viruses. VSV-G pseudotyped viruses were made by co-transfecting 293T cells with VSV-G plasmid and following plasmids (WT, ΔVpu, L30E and L30E-Δvpu). Progeny pseudovirions were harvested at 48 h, clarified by centrifugation (2000 rpm for 5 min), filtered through 0.45 µm pore size syringe filter and tested for infectivity in GHOST-CD4-CXCR4 cells. (A) Viruses were serially diluted and equal amount of supernatant was added on GHOST-CD4-CXCR4 cell plated in 96-well plate (10,000 cells/well). As positive infectivity control, pNL4.3 virus without VSV-G was added and as negative control pNL-AD8 virus without VSV-G was added on GHOST-CD4-CXCR4 cells. 48 h post-infection, GHOST cells were fixed with acetone-methanol (1∶1 ratio) and immunostained for p24 antigen. Blue cells represent focus forming units (FFU) or infectious units. (B) Blue cells were counted as infectious units, their average was calculated per millilitre and plotted in a graph. (TIF) [file pone.0061388.s001.tif]
