# Supplementary figures and images for: Expression of Concern: Evidence that Vpu Modulates HIV-1 Gag-Envelope Interaction towards Envelope Incorporation and Infectivity in a Cell Type Dependent Manner
Source: PLoS One. 2024 Mar 26;19(3):e0301303. doi: 10.1371/journal.pone.0301303 (PMC10965049; doi:10.1371/journal.pone.0301303)

WT

d-Vpu

WT

d-Vpu

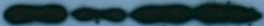

NP2

GHOST

Supplement: S1 File — (ZIP) [file pone.0301303.s001.zip › PONE_Zip/Fig 2/NP2-Ghost blot.pdf]

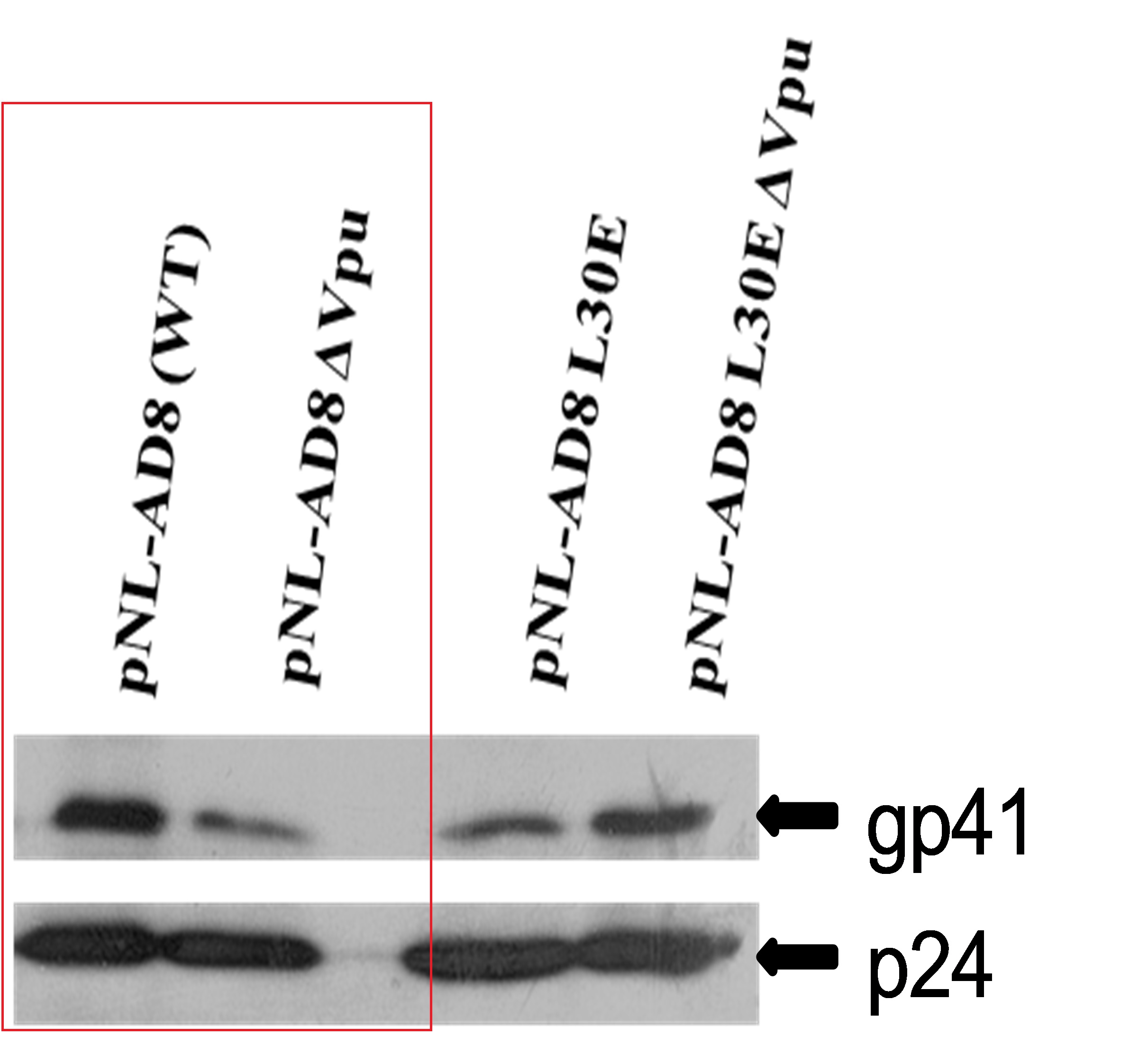

Supplement: S1 File — (ZIP) [file pone.0301303.s001.zip › PONE_Zip/Fig 2/Fig 2_query 3.jpg]

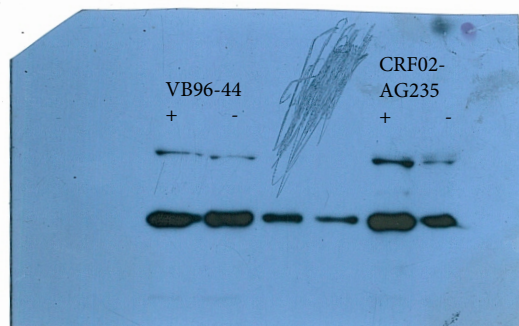

Supplement: S1 File — (ZIP) [file pone.0301303.s001.zip › PONE_Zip/Fig 6/Fig 6B right panel.pdf]

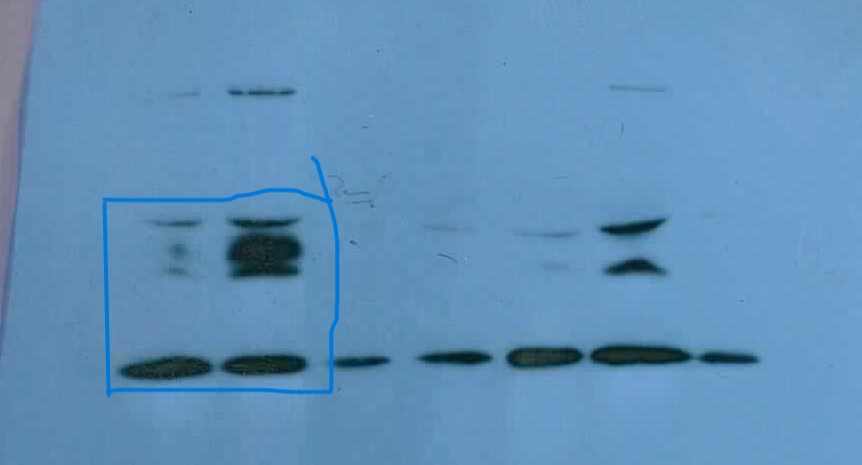

Supplement: S1 File — (ZIP) [file pone.0301303.s001.zip › PONE_Zip/Fig 6/Fig 6E VB96-44.jpg]

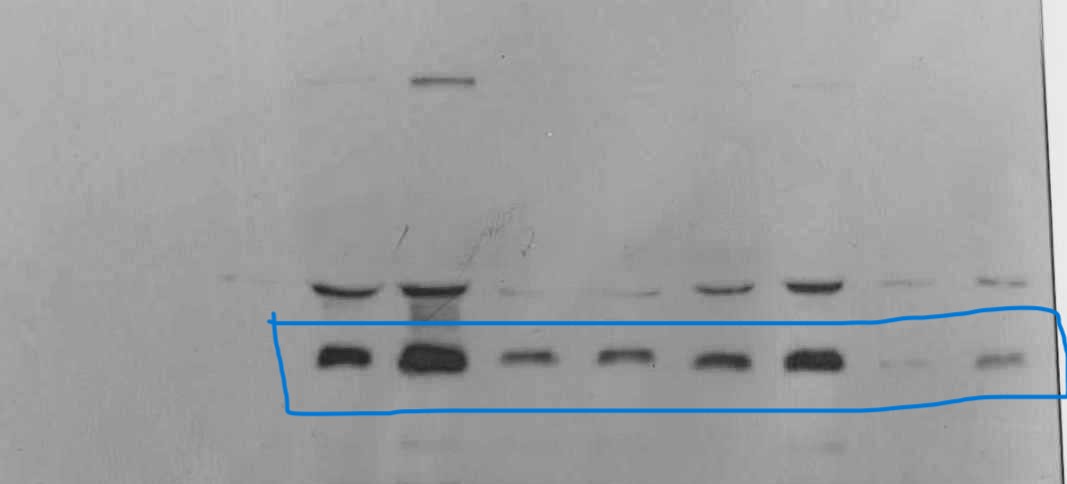

Supplement: S1 File — (ZIP) [file pone.0301303.s001.zip › PONE_Zip/Fig 6/Fig 6E gp41.jpg]

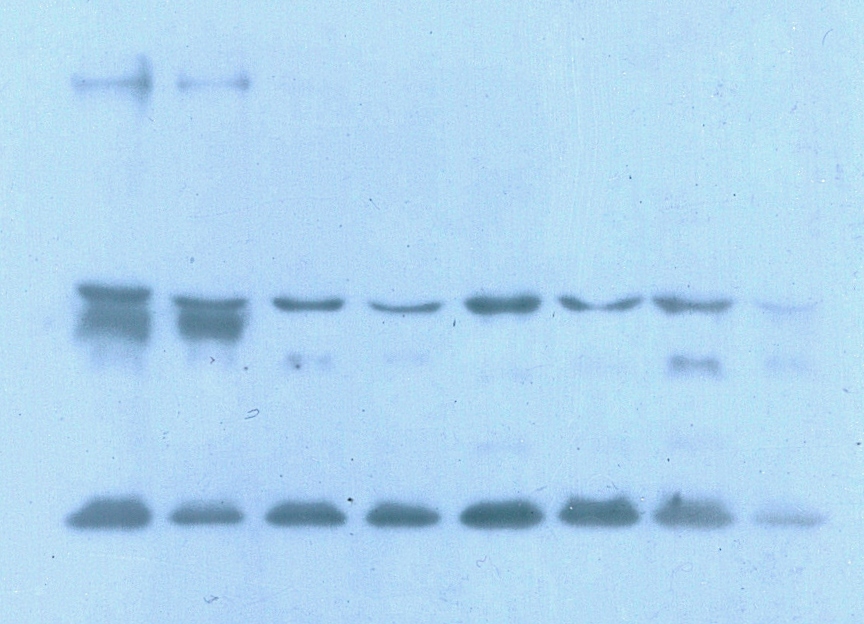

Supplement: S1 File — (ZIP) [file pone.0301303.s001.zip › PONE_Zip/Fig 6/Fig 6B left panel.jpg]

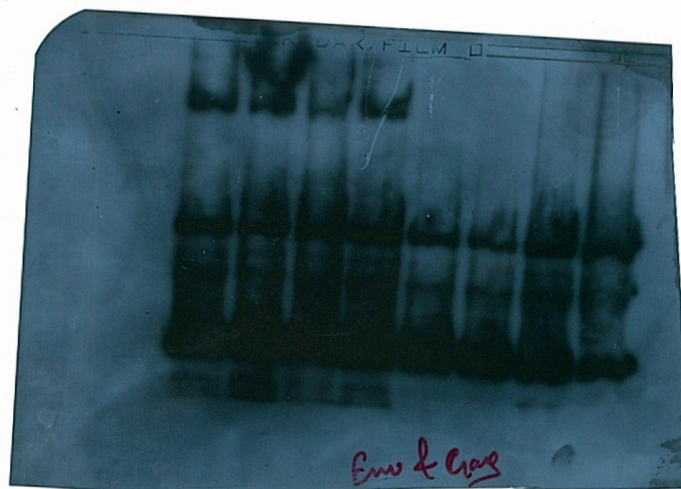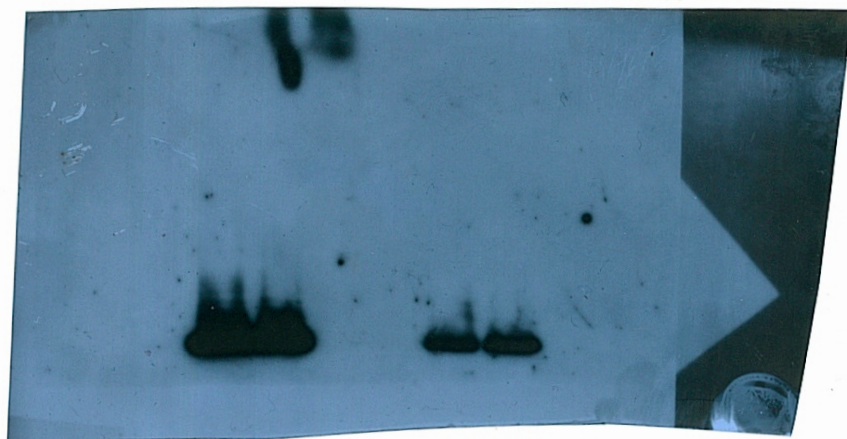

Supplement: S1 File — (ZIP) [file pone.0301303.s001.zip › PONE_Zip/Fig 1/Fig_1B.pdf]

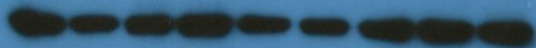

Supplement: S1 File — (ZIP) [file pone.0301303.s001.zip › PONE_Zip/Fig 8/Fig 8B B-actin blot2.pdf]

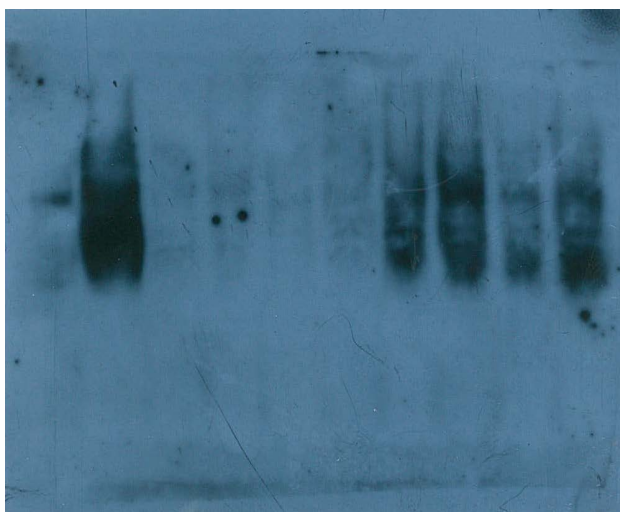

Supplement: S1 File — (ZIP) [file pone.0301303.s001.zip › PONE_Zip/Fig 8/Fig 8B BST2.pdf]

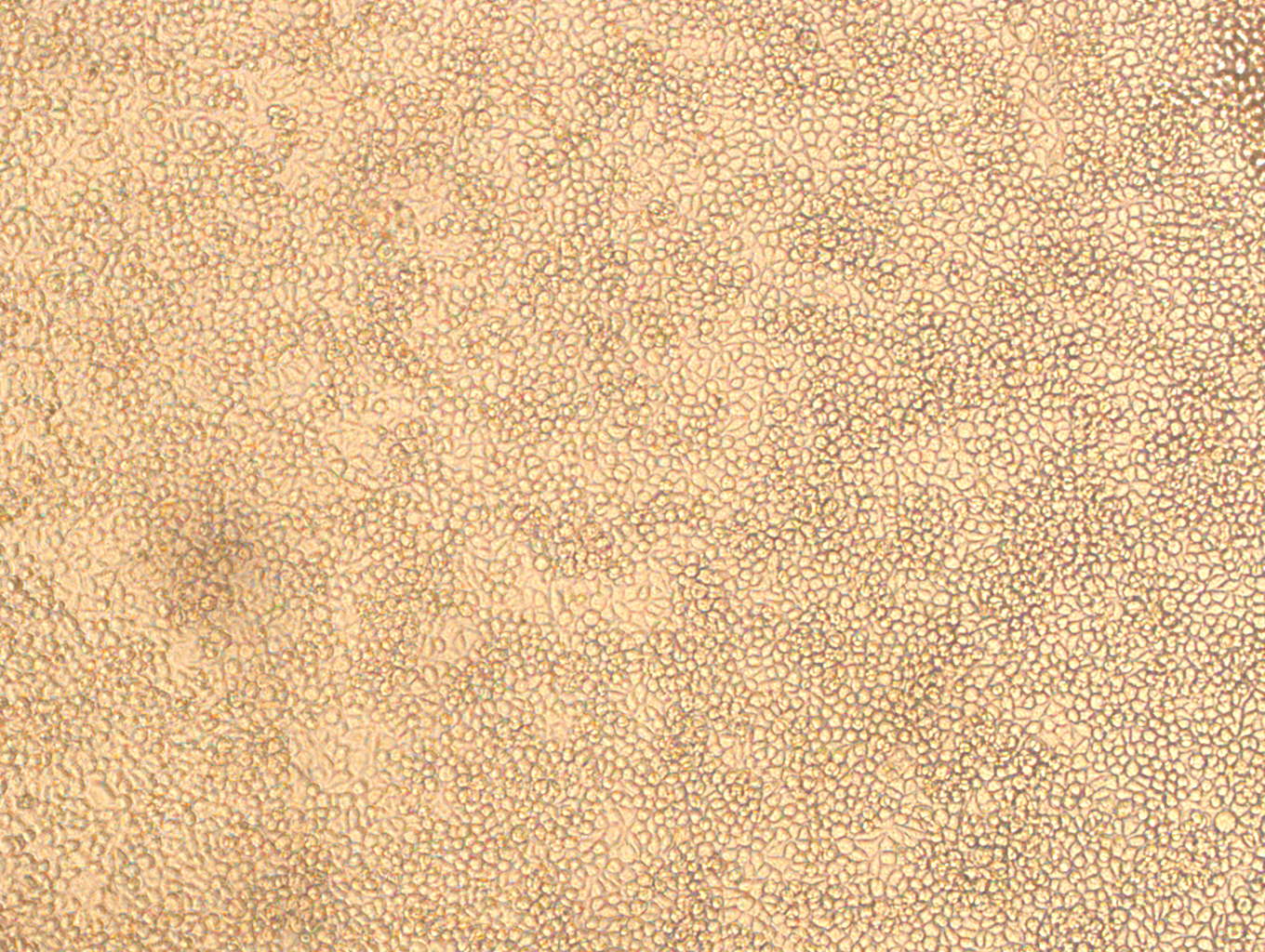

Supplement: S1 File — (ZIP) [file pone.0301303.s001.zip › PONE_Zip/Fig 4/Fig 4B_images/6Dy P.A DV.jpg]

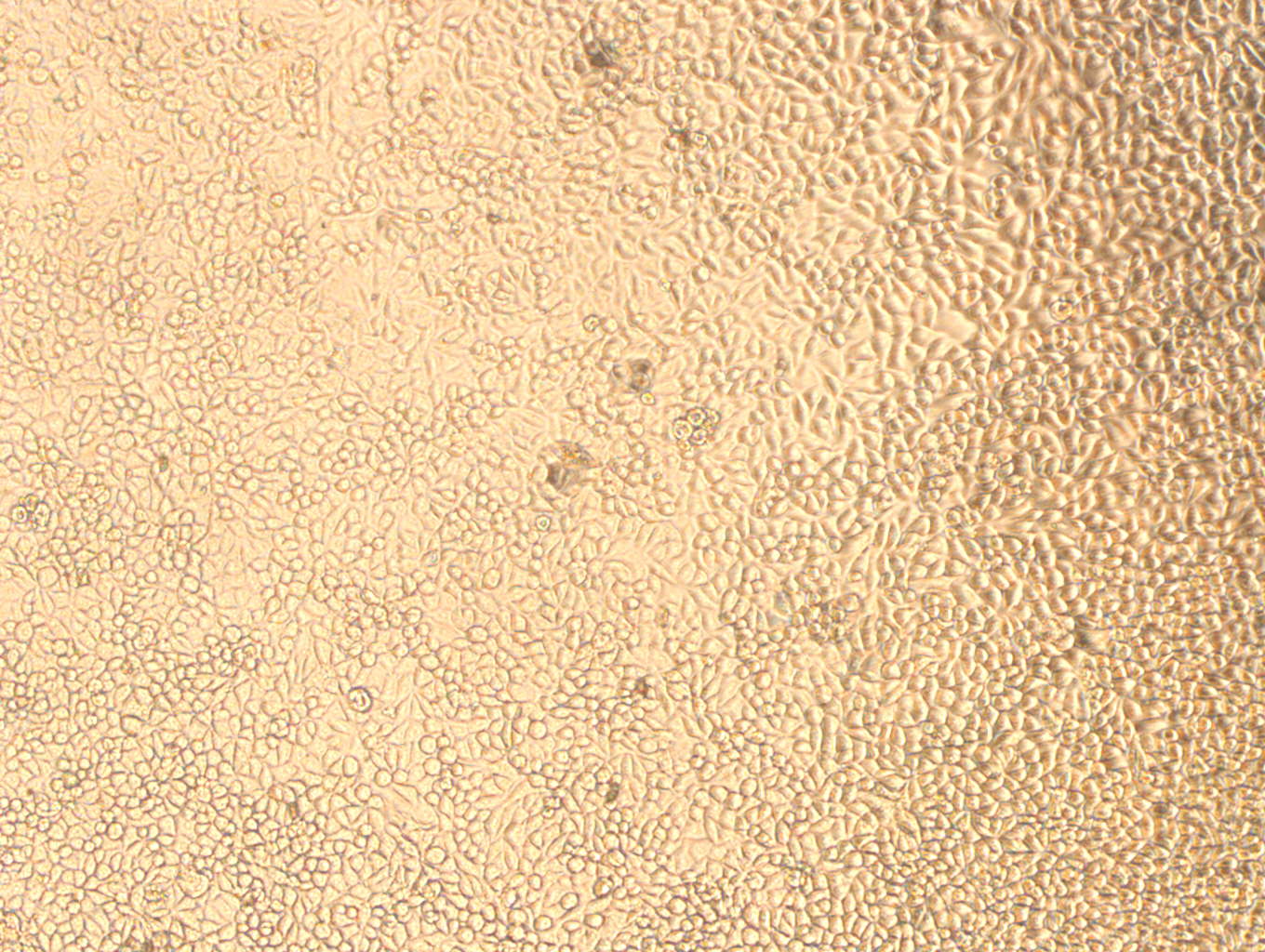

Supplement: S1 File — (ZIP) [file pone.0301303.s001.zip › PONE_Zip/Fig 4/Fig 4B_images/0Dy P.A L30E DV.jpg]

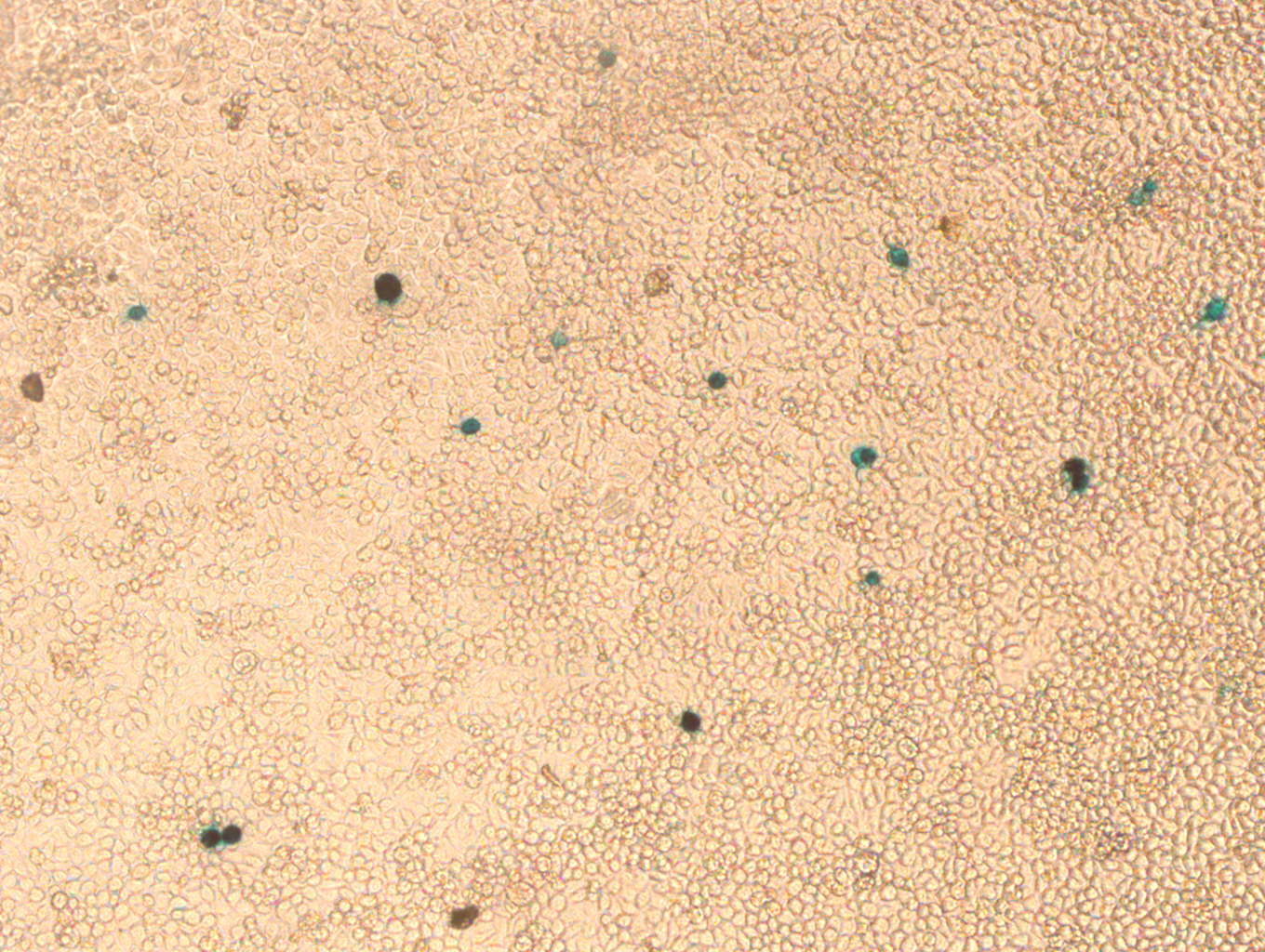

Supplement: S1 File — (ZIP) [file pone.0301303.s001.zip › PONE_Zip/Fig 4/Fig 4B_images/12Dy P.A DV.jpg]

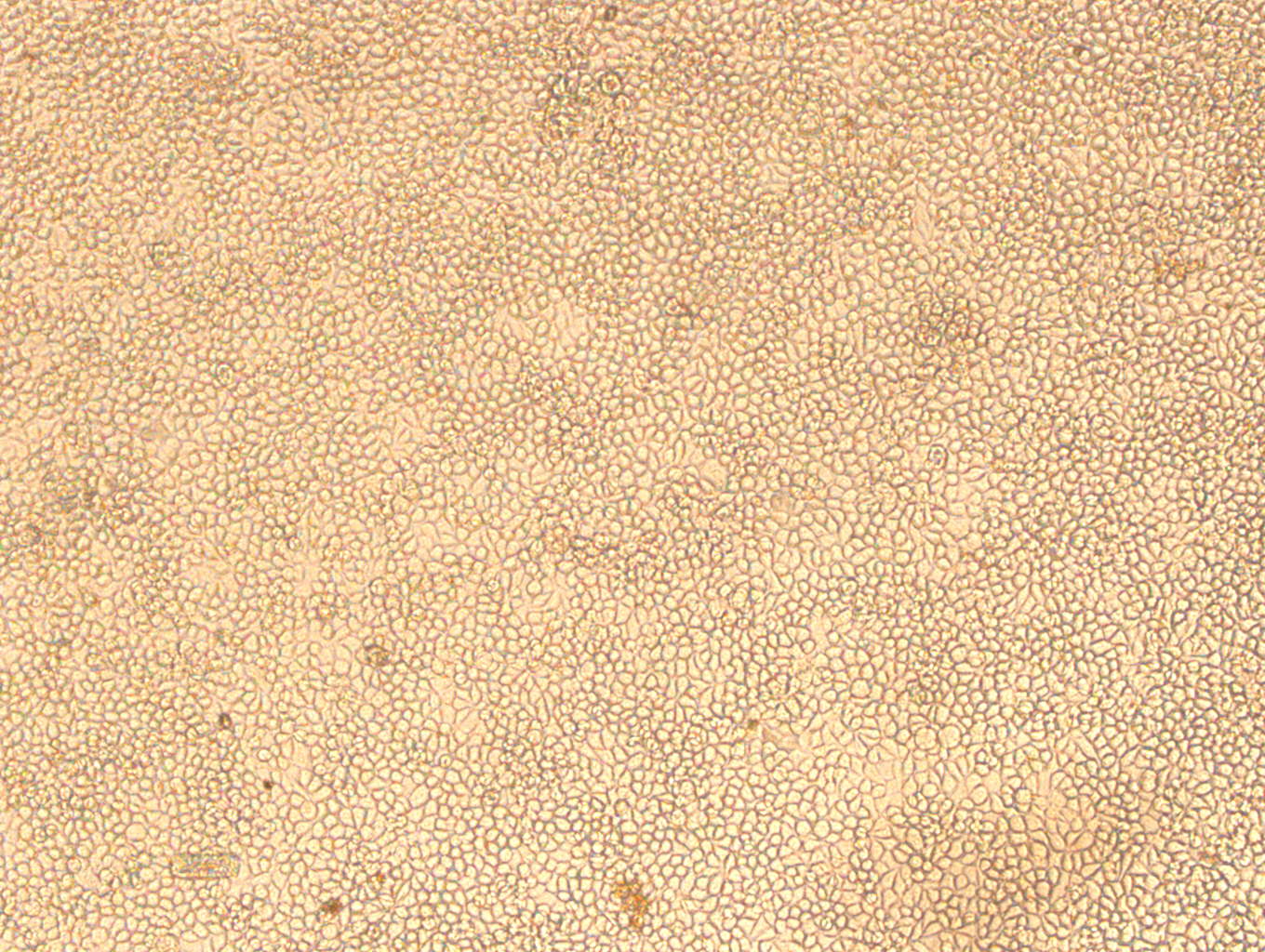

Supplement: S1 File — (ZIP) [file pone.0301303.s001.zip › PONE_Zip/Fig 4/Fig 4B_images/0Dy P.A DV.jpg]

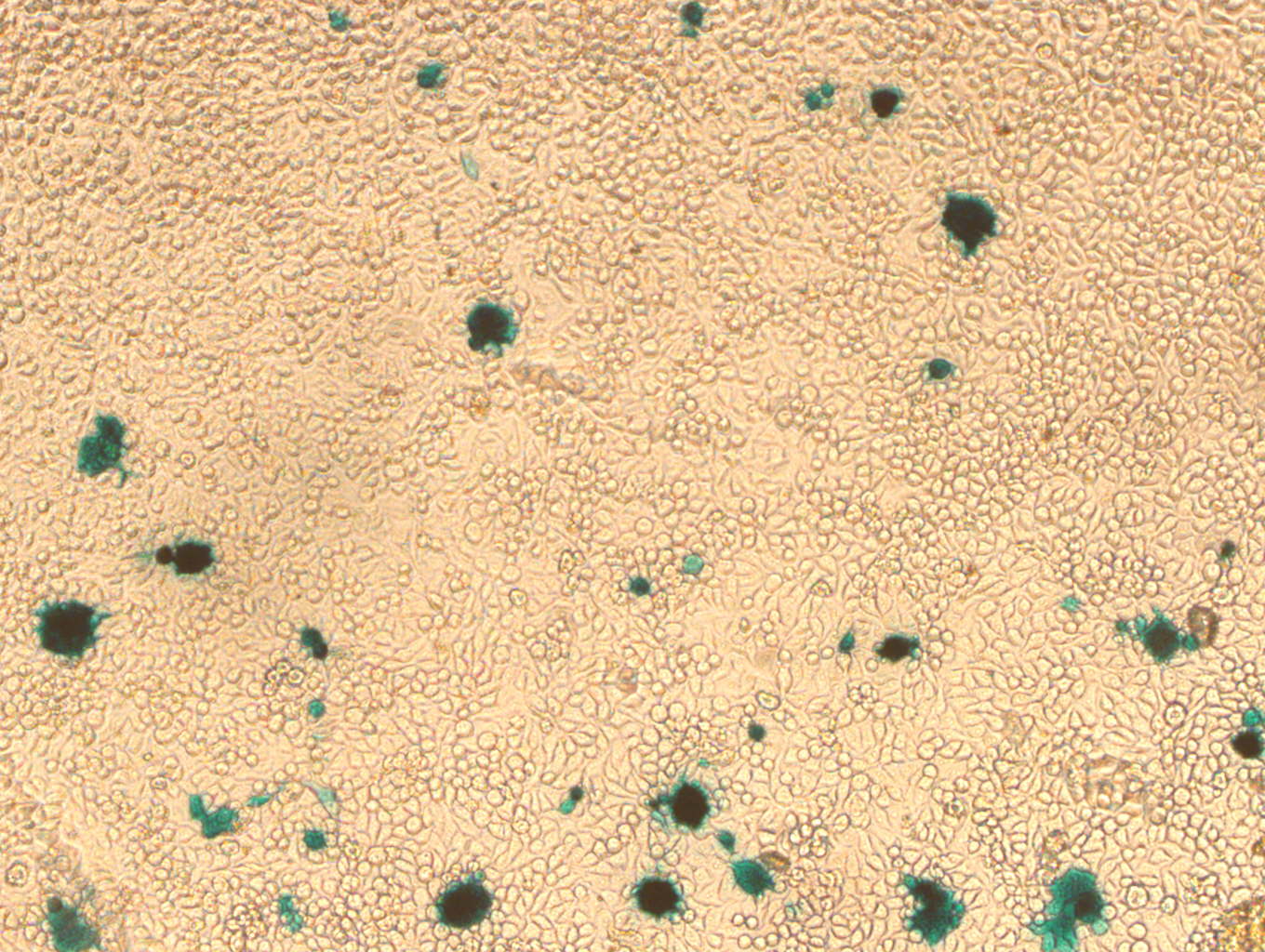

Supplement: S1 File — (ZIP) [file pone.0301303.s001.zip › PONE_Zip/Fig 4/Fig 4B_images/6Dy P.A WT.jpg]

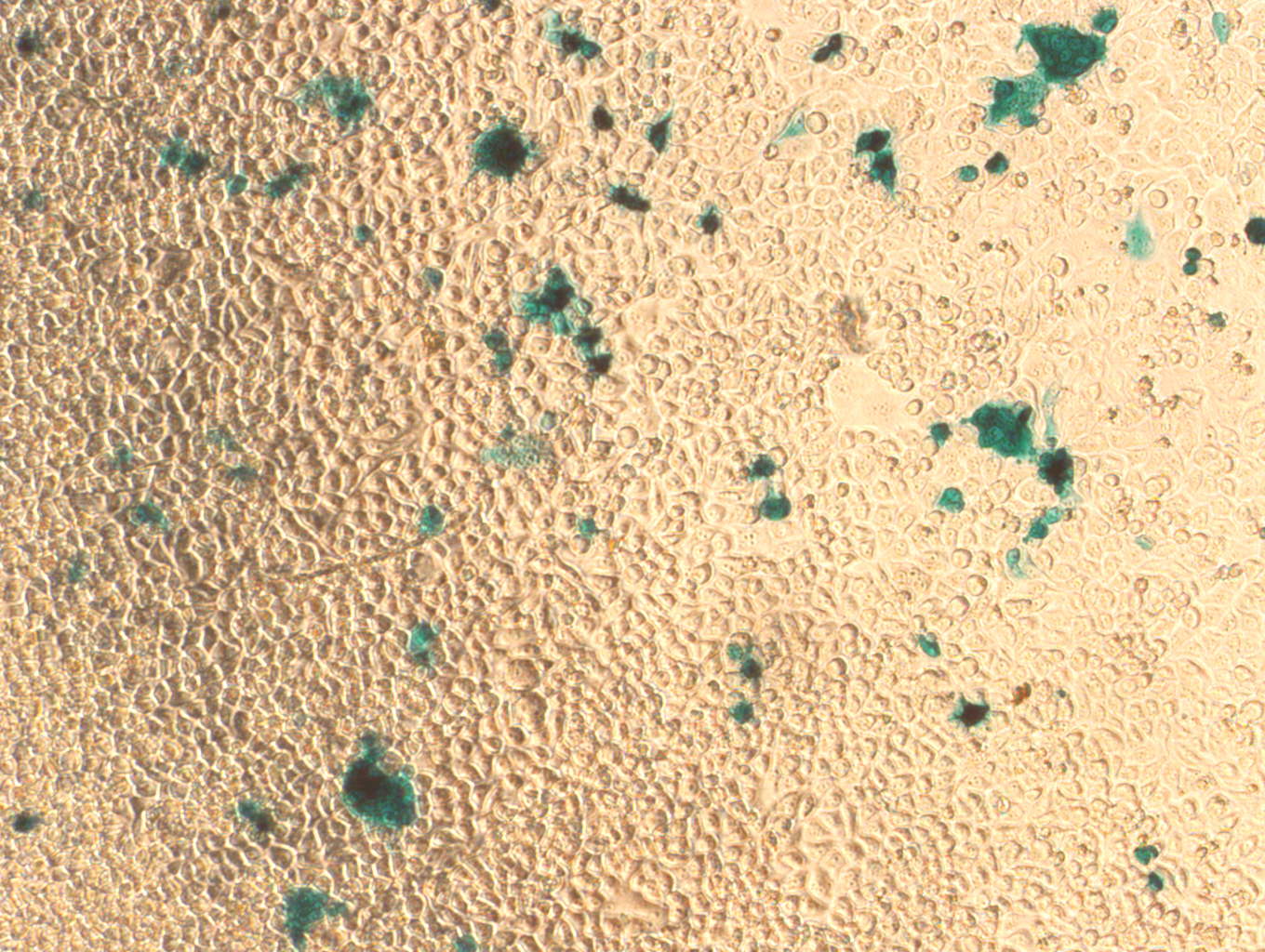

Supplement: S1 File — (ZIP) [file pone.0301303.s001.zip › PONE_Zip/Fig 4/Fig 4B_images/12Dy P.A WT.jpg]

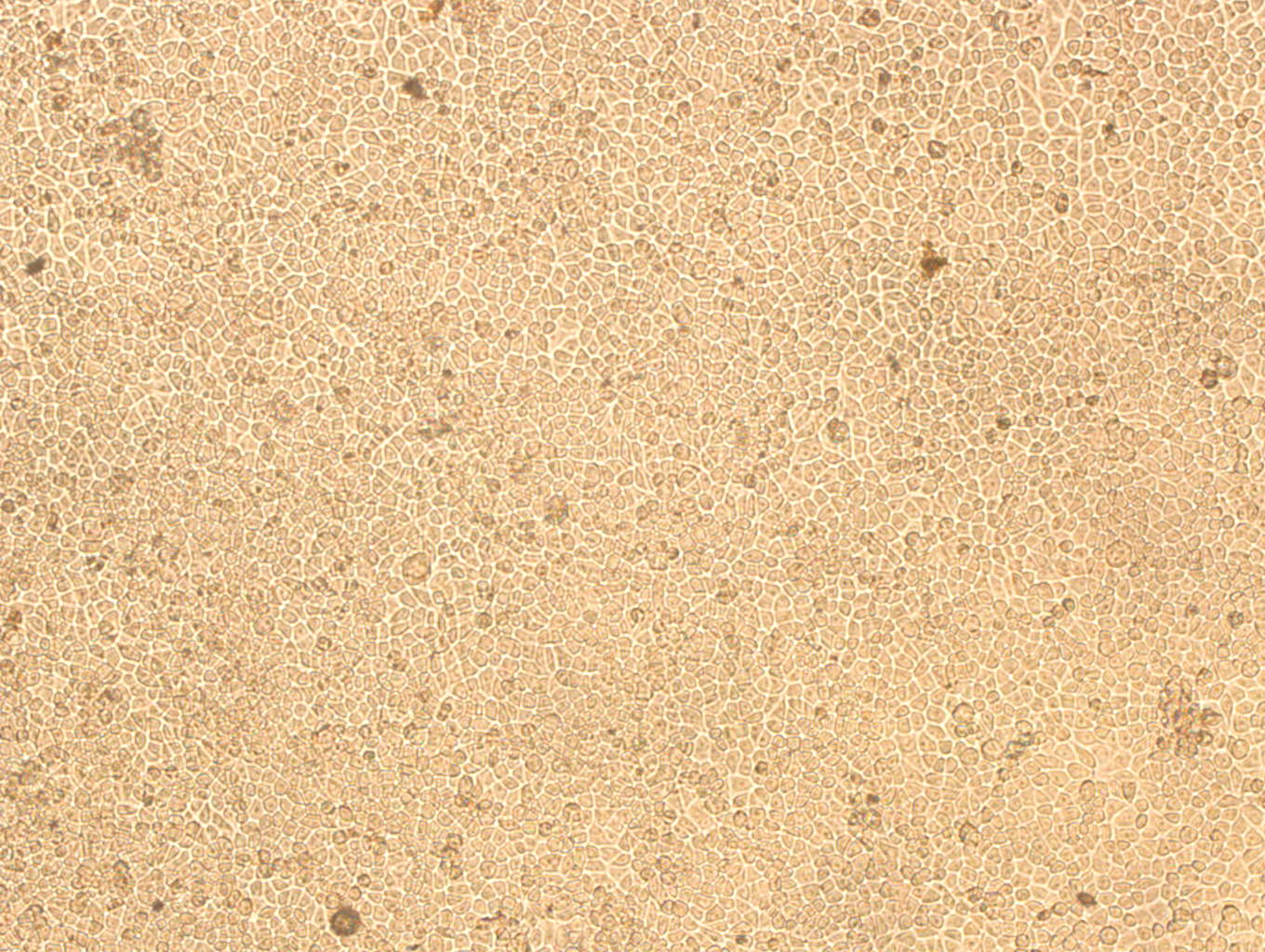

Supplement: S1 File — (ZIP) [file pone.0301303.s001.zip › PONE_Zip/Fig 4/Fig 4B_images/0Dy P.A WT.jpg]

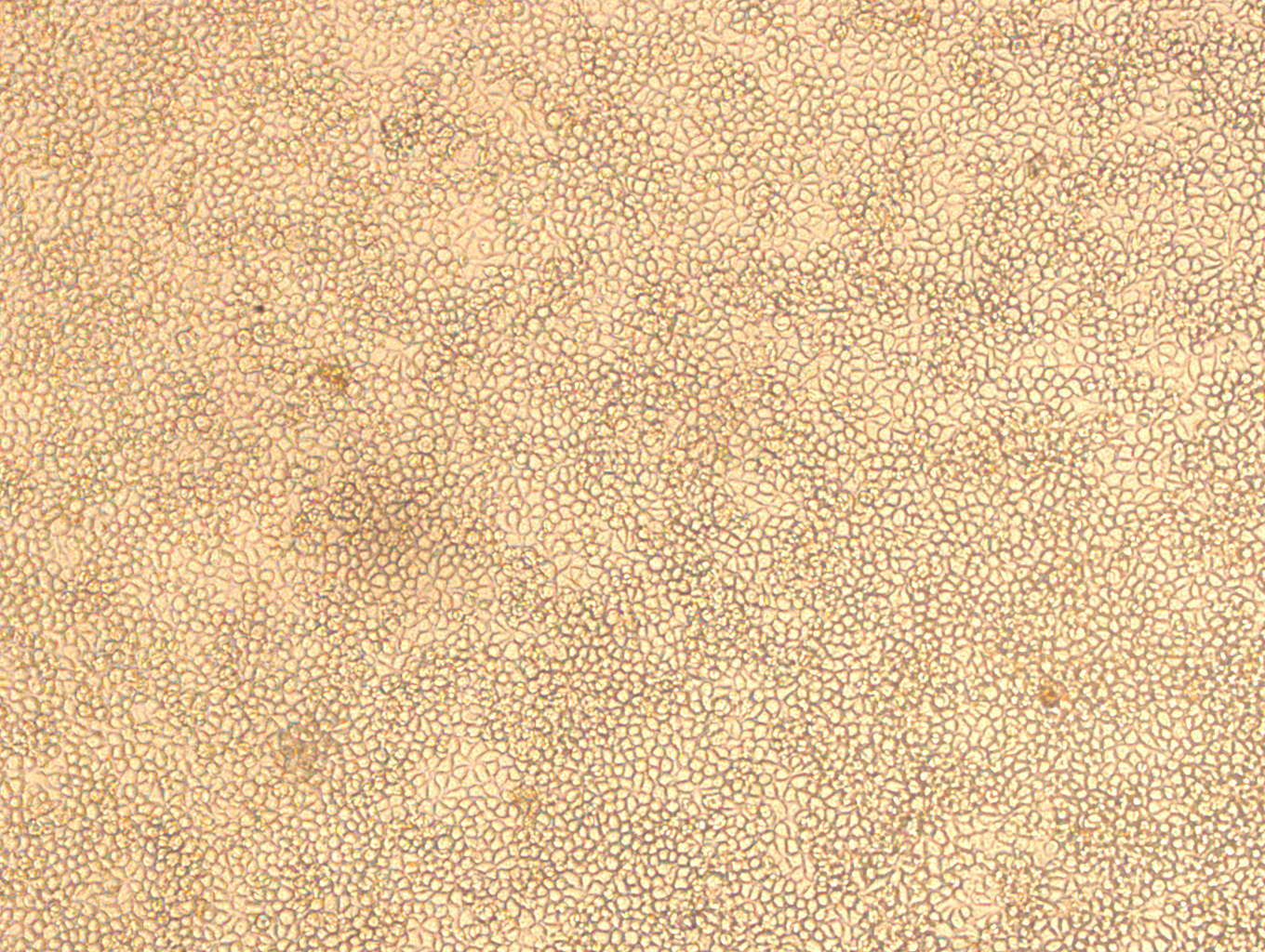

Supplement: S1 File — (ZIP) [file pone.0301303.s001.zip › PONE_Zip/Fig 4/Fig 4B_images/6Dy P.A DV L30E.jpg]

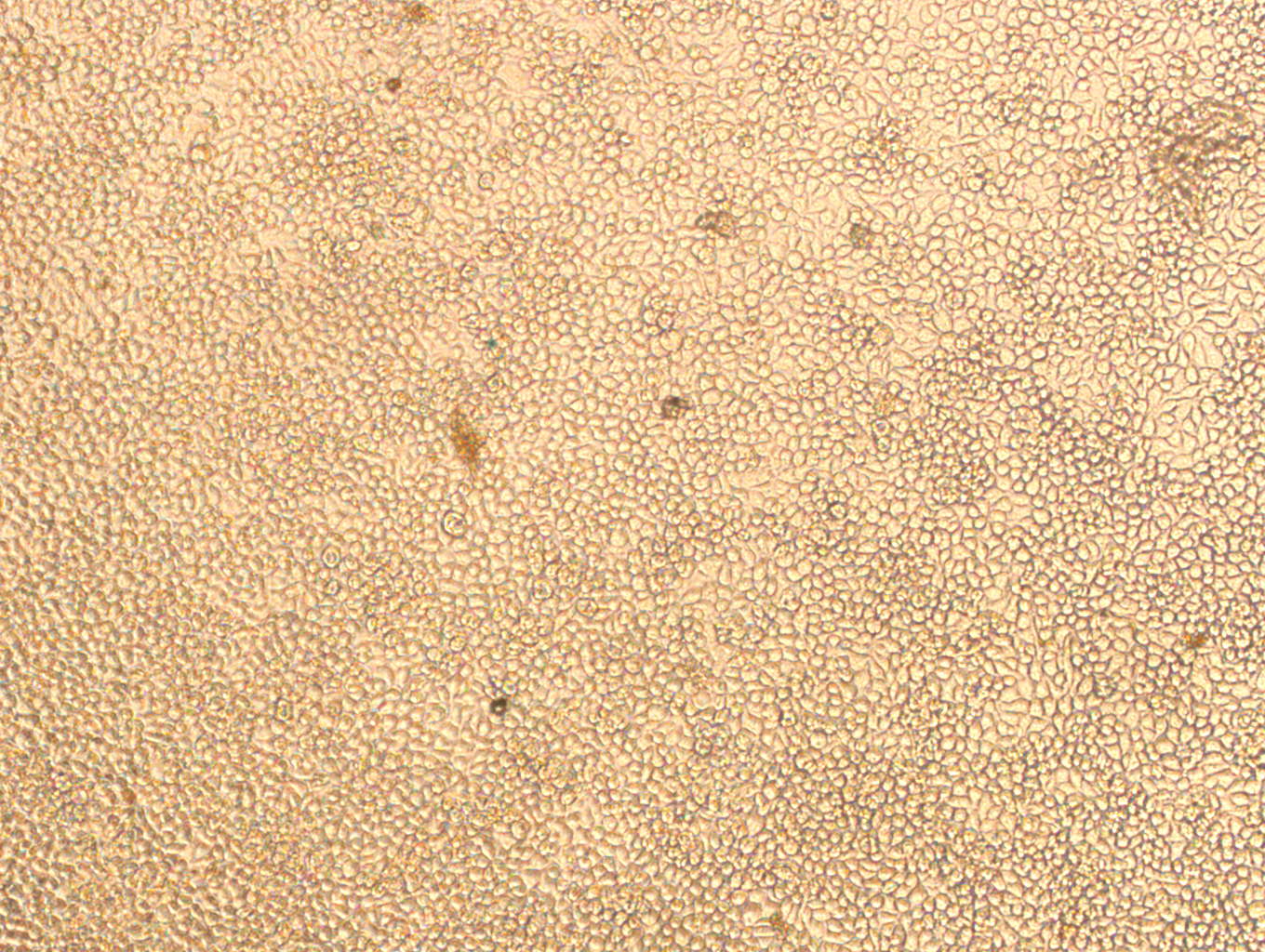

Supplement: S1 File — (ZIP) [file pone.0301303.s001.zip › PONE_Zip/Fig 4/Fig 4B_images/12Dy P.A L30E.jpg]

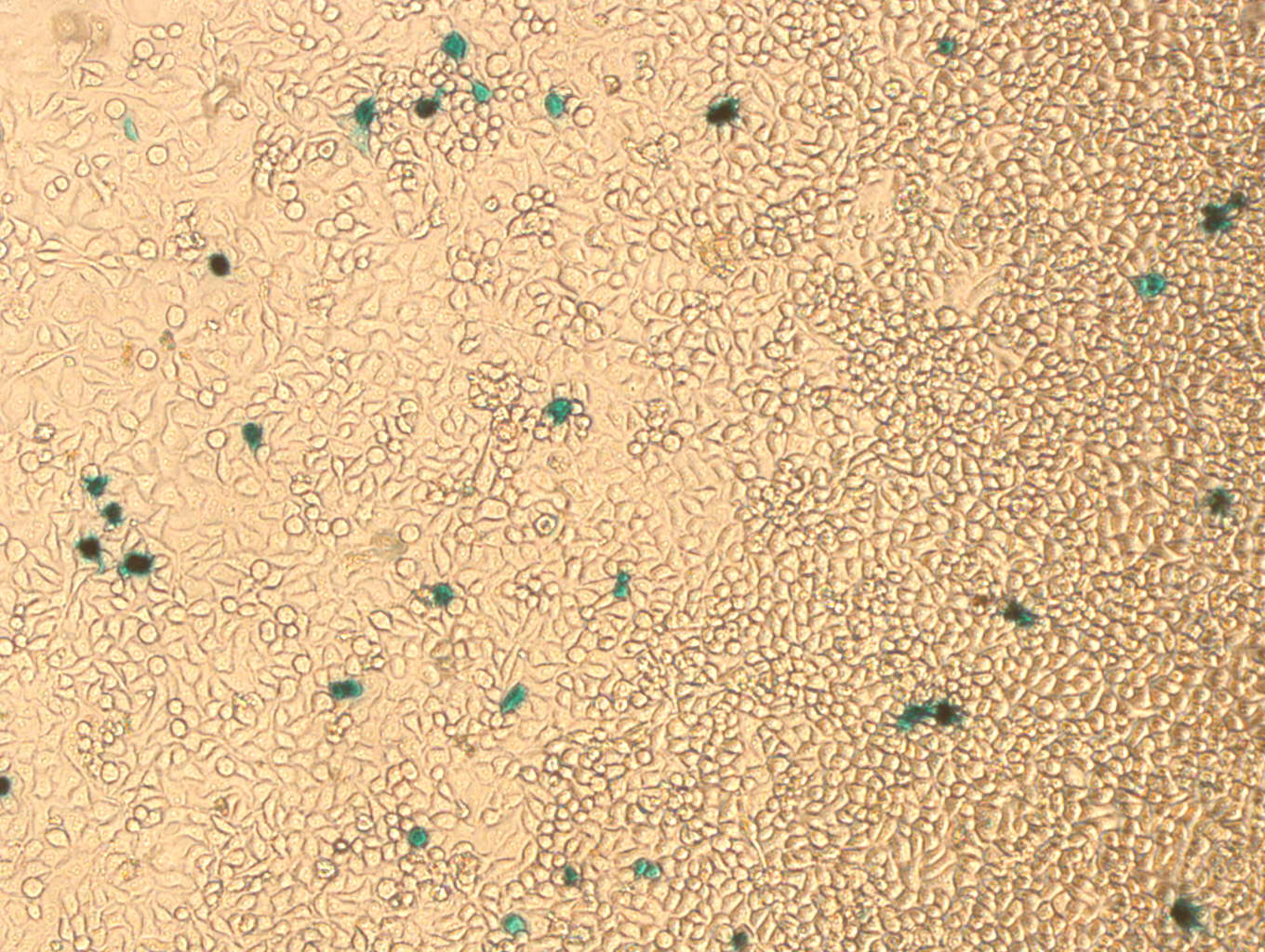

Supplement: S1 File — (ZIP) [file pone.0301303.s001.zip › PONE_Zip/Fig 4/Fig 4B_images/12Dy P.A DV L30E.jpg]

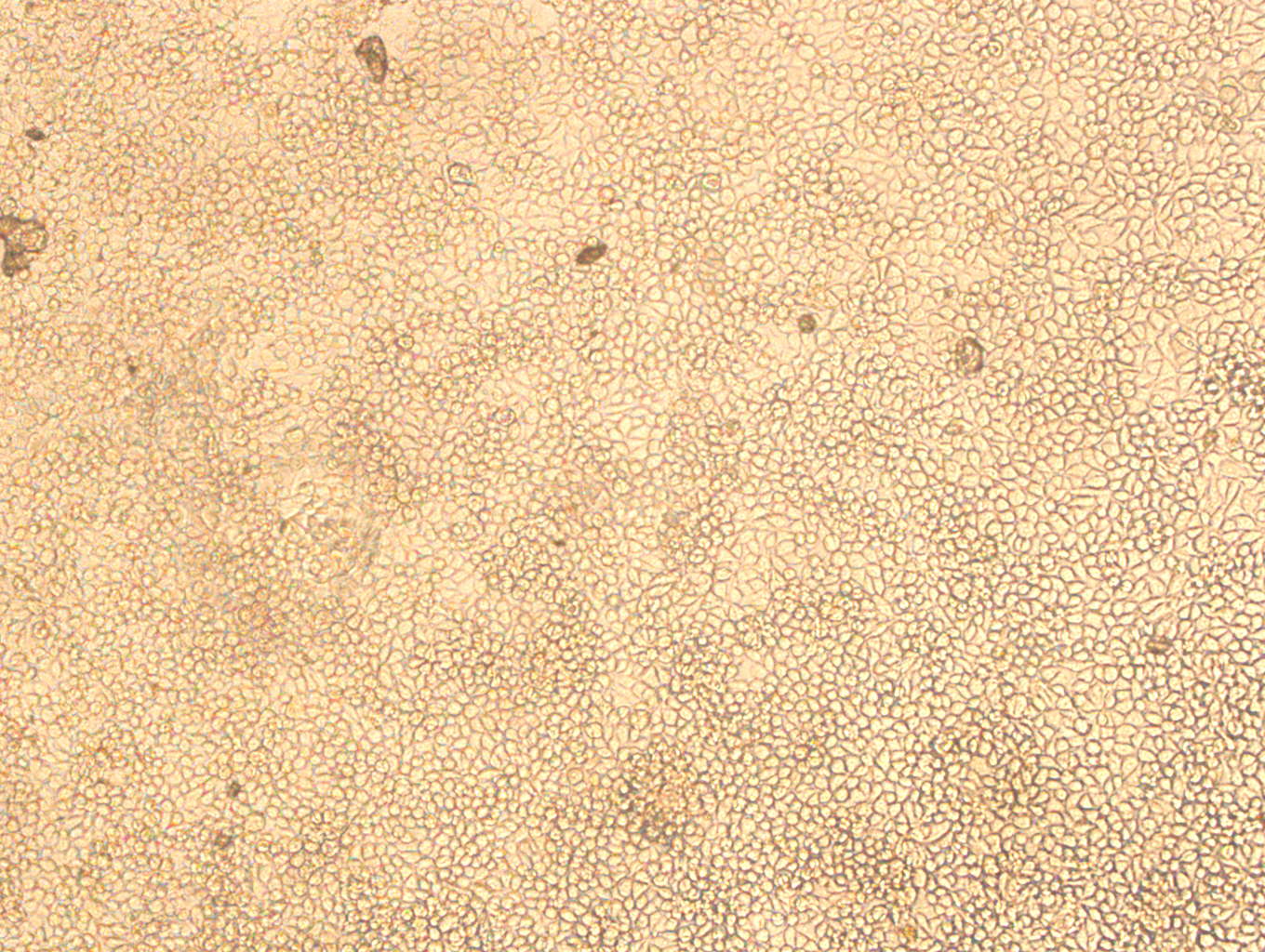

Supplement: S1 File — (ZIP) [file pone.0301303.s001.zip › PONE_Zip/Fig 4/Fig 4B_images/0Dy P.A L30E.jpg]

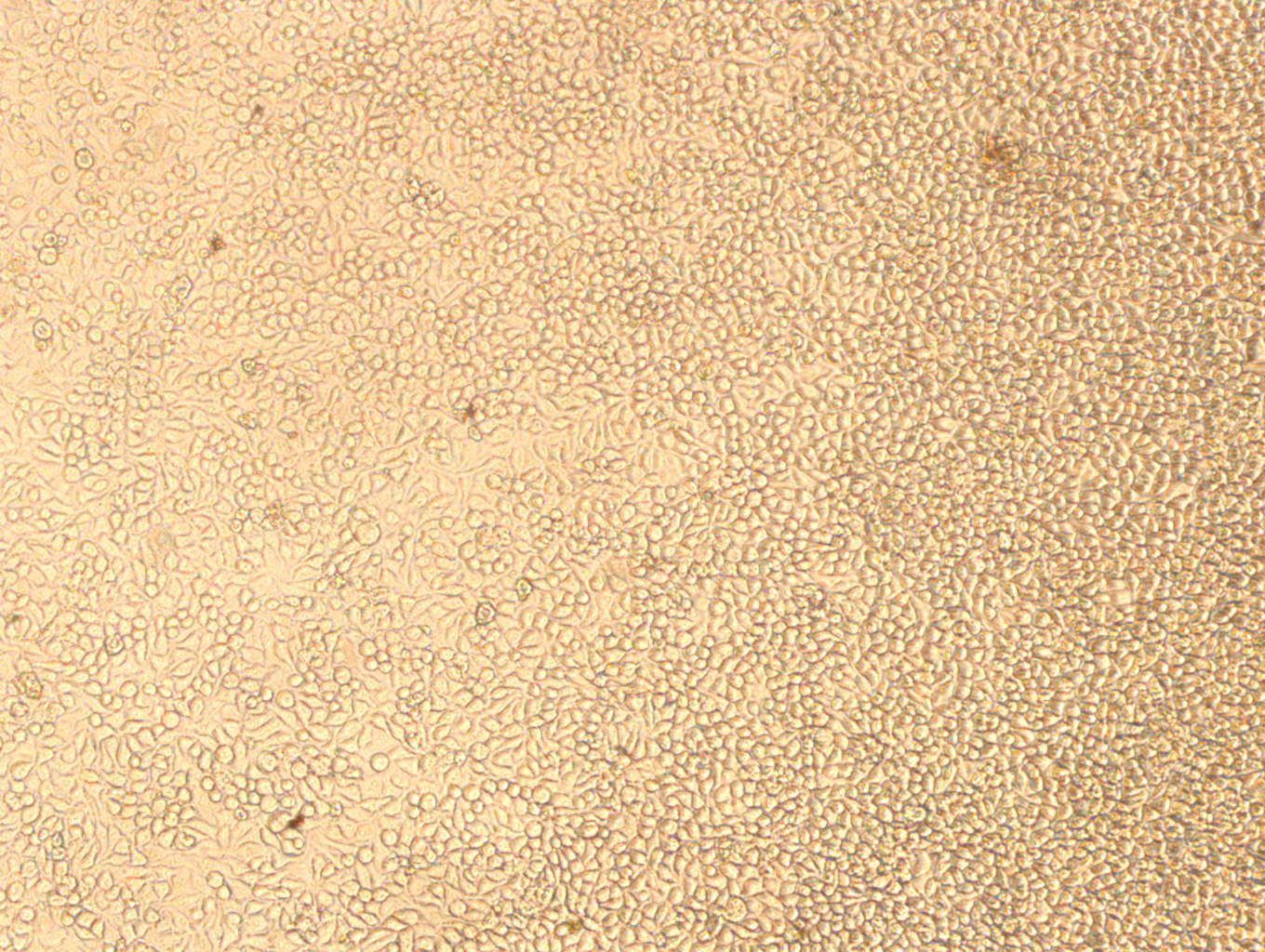

Supplement: S1 File — (ZIP) [file pone.0301303.s001.zip › PONE_Zip/Fig 4/Fig 4B_images/6Dy P.A L30E.jpg]

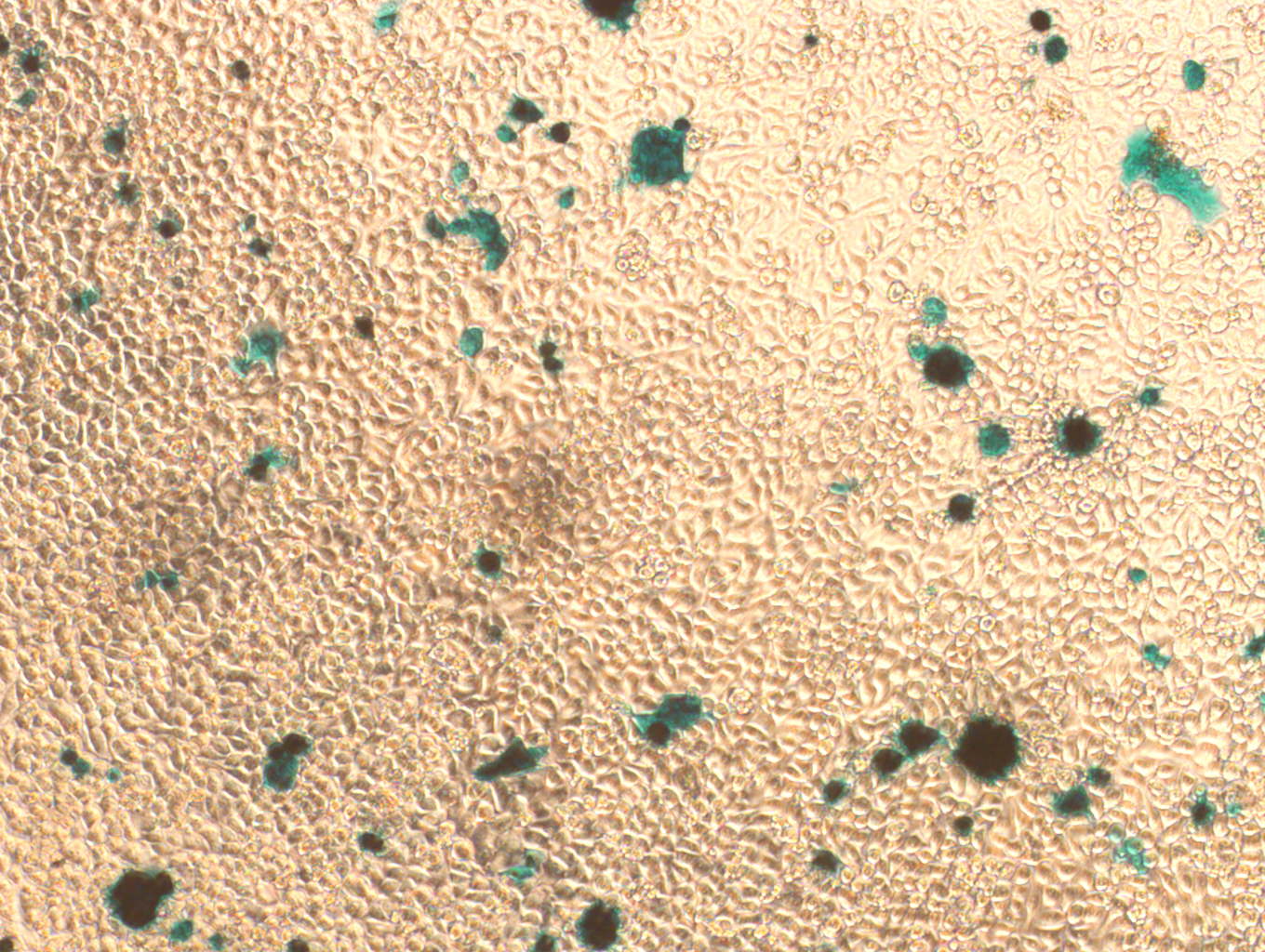

Supplement: S2 File — (JPG) [file pone.0301303.s002.jpg]

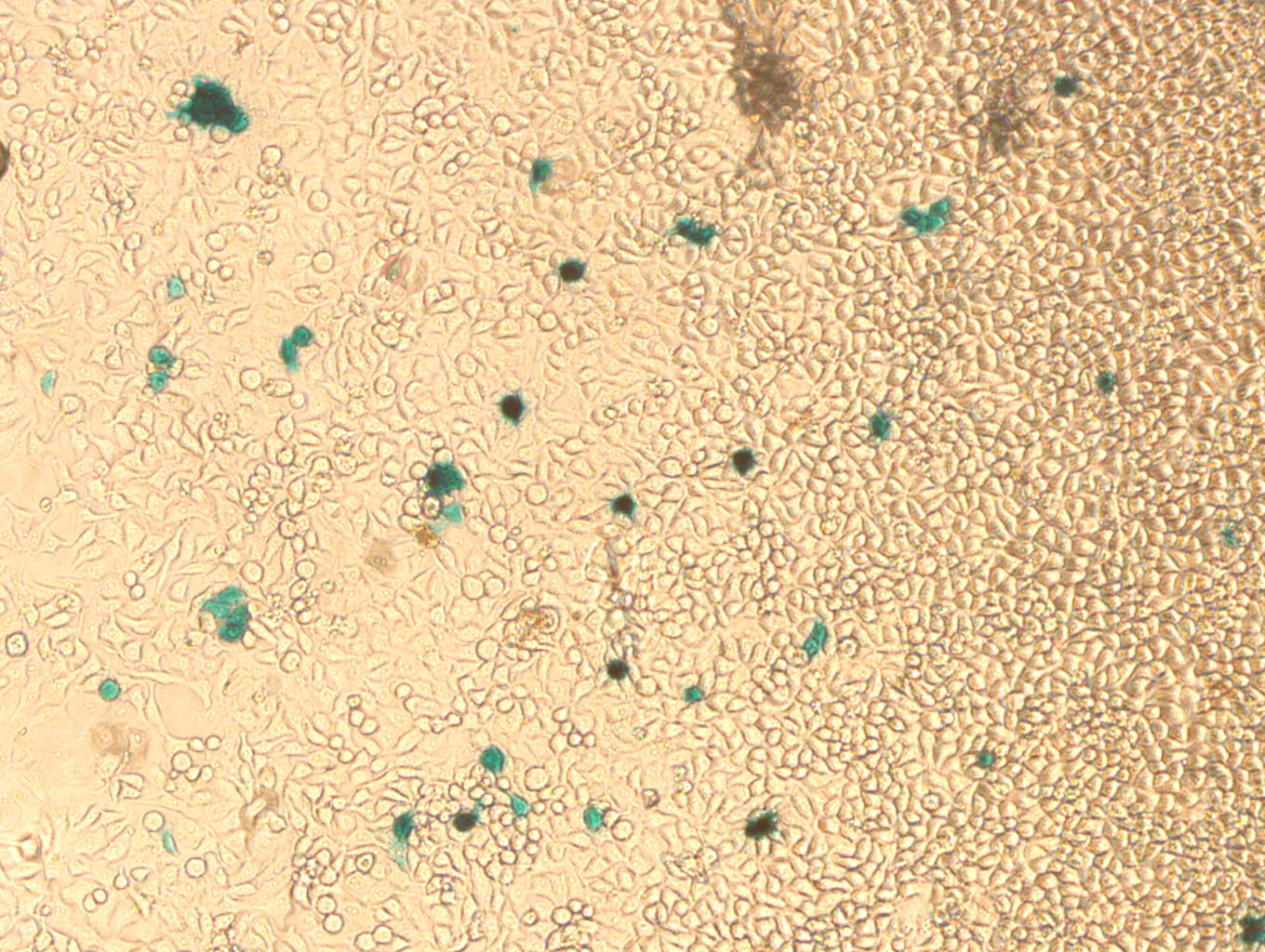

Supplement: S3 File — (JPG) [file pone.0301303.s003.jpg]
